# Preparation and Electrochemical Performance Investigation of Nano-Silicon-Enhanced Graphite Materials Based on Mechanical Grinding Process

**DOI:** 10.3390/nano16140889

**Published:** 2026-07-20

**Authors:** Limeng Lei, Jian Yang, Dongran Song, Runxin Chen, Liqing Liao

**Affiliations:** 1 School of Automation, Central South University, Changsha 410083, China; 234602064@csu.edu.cn (L.L.); songdongran@csu.edu.cn (D.S.); zdh-dqkz@csu.edu.cn (L.L.); 2 Institute of New Energy Control and Industrial Intelligence, Central South University, Changsha 410083, China; jian.yang@csu.edu.cn

**Keywords:** lithium-ion batteries, nano-silicon-enhanced graphite anode material, wet grinding, nano-silicon

## Abstract

Lithium-ion batteries are widely used in digital, power and energy storage fields due to their high capacity and high cycle life advantages. This paper systematically screens the formulation system and designs a high-efficiency production line that can effectively improve production efficiency, reduce production costs, and lower energy consumption per unit product. The produced nano-silicon-enhanced graphite anode material has excellent performance. The selection of silicon raw materials, types of solvents, types of dispersants, and grinding processes is studied to investigate the influence of these four factors on the wet grinding process for preparing nano-silicon. Finally, metal silicon obtained by air flow pulverization is selected as the raw material, isopropanol is used as the solvent, FA01 (carboxylic acid type) is used as the dispersant, and a two-stage wet grinding process is adopted to prepare the nano-silicon dispersion solution. Zirconia beads of 0.5 mm and 0.2 mm size are used as the grinding media for the first and second stages, respectively, with filling rates of 80% and 90%, respectively. The final prepared nano-silicon dispersion is stable in dispersion and has a narrow particle size distribution. The nano-silicon dispersion solution and the multi-walled carbon nanotube dispersion solution are mechanically ground and mixed using a sand mill. At the same time, the multi-walled carbon nanotubes are coated with the nano-silicon. Then, artificial graphite is added for compounding. Finally, through spray drying, the Si@MWCNTs@graphite (SMG) nano-silicon-enhanced graphite negative electrode material is prepared. The SMG nano-silicon-enhanced graphite negative electrode material with a silicon content of 2% has a first Coulomb efficiency of up to 84.32%.

## 1. Introduction

With the advancement of industrial technology and economic level, the energy and environmental crises have become increasingly prominent. The excessive exploitation and utilization of resources such as oil and coal have not only caused global environmental problems, but also faced the serious issue of resource depletion. Therefore, the development and application of new green, environmentally friendly, and renewable energy and materials is the inevitable path to achieving sustainable development.

At the end of the 20th century, the emergence of lithium-ion batteries opened a brand-new door for the development of new energy and new materials. Lithium-ion batteries have features such as high voltage, high specific capacity, long cycle life, low self-discharge, fast charging, small size, no memory effect, and portability. They have been widely used in portable electronic products such as mobile phones and laptops. Currently, graphite is the most commonly used negative electrode material in the industrial field. This material has a certain theoretical specific capacity, specifically 372 mAh/g. As a core component of lithium-ion batteries, its theoretical capacity is relatively low, greatly restricting the application of lithium-ion batteries in automotive power. Therefore, finding a new type of high-specific-capacity and long-cycle negative electrode material can significantly improve the performance of the battery, including the improvement in energy and power density as well as safety. High efficiency and low-cost production are extremely important. New negative electrode materials should meet certain requirements: (1) a high theoretical specific capacity and low lithium alloying potential; (2) good electronic and ionic conductivity; (3) minimal volume expansion during lithiation; and (4) low cost and ease of large-scale production. In addition, when designing negative electrode materials, attention should be focused on the material structure, reducing the specific surface area, and increasing the compactness, energy density, etc. as much as possible. Currently, several types of promising negative electrode materials include hard carbon [1,2], tin-based materials [3,4], silicon-based materials [5,6,7], etc. These materials can meet the above requirements, such as theoretical specific capacity, but do not have high Coulomb efficiency, and the capacity decays at a relatively fast rate. Among them, negative electrode materials have a continuously expanding application range, but require complex processing techniques and high manufacturing costs, resulting in high prices, and cannot be mass-produced or widely applied to automotive power [8,9,10,11]. Therefore, finding a new process to reduce the production cost of negative electrode materials and improve their production efficiency is the key to solving the range problem of lithium-ion batteries in automotive power applications.

In lithium-ion batteries, the main components include the positive and negative electrodes, the separator, and the electrolyte. Depending on the application, the size and shape of lithium-ion batteries vary. There are mainly four types of batteries: cylindrical batteries, pouch batteries, button batteries, and square batteries.

Silicon belongs to the alloy-type anode. The lithium storage mechanism of silicon as an anode material is that, during battery charging, silicon forms a series of alloys with lithium [12,13,14,15]. Regarding this, many scholars have conducted extensive research. Reference [16] found that, when the particle size of silicon is less than 150 nm, the silicon particles do not rupture during the first lithium insertion process. Therefore, researchers need to prepare at least a nano-film, nanowire, nanoparticle or nano-porous material with a dimension lower than 150 nm to inhibit the structural damage caused by volume expansion. Reference [17] used cluster deposition technology, using pre-deposited Cu nanoparticles to assemble a film as a growth template, successfully preparing a porous Si/Cu composite amorphous film. Reference [18] used stainless steel as the substrate and gold as the catalyst, and used the VLS method to prepare silicon nanowires with an average diameter of 89 nm. This material can still maintain its nanowire morphology after cycling and has stable cycling performance.

In conclusion, by nanocrystallizing the silicon anode, the damage caused by volume changes can be alleviated to a certain extent, thereby improving the electrochemical cycling performance of the silicon anode. Secondly, when the silicon is nanocrystallized and used as the anode, it is beneficial to expand the contact area with the electrolyte, facilitating the efficient transmission of lithium ions and achieving an increase in rate performance. However, at the same time, a large amount of SEI film will be generated, reducing the initial Coulomb efficiency of the silicon anode. At the same time, the nanoscale silicon anode material also has problems such as high cost and low production efficiency that need to be solved.

Silicon nanocrystallization is a modification method that refines silicon materials to the nanoscale through various processes such as physical and chemical methods, and constructs characteristic microstructures [19]. It includes mainstream approaches like ball milling, magnesium thermal reduction, vapor deposition, and etching [20]. Nanocrystallization can shorten the distance of lithium-ion transmission, slow down the volume expansion during charging and discharging, and effectively improve the rate and cycle performance of silicon negative electrodes [21]. However, the high specific surface area is prone to causing side reactions in the electrolyte, and in practical applications, it is often combined with carbon coating for collaborative optimization [22]. The silicon sodiumization method can reduce problems such as volume expansion and improve the performance of the negative electrode material. However, the issues of poor conductivity and unstable SEI film still exist. Among the various surface coating strategies reported for silicon anodes, carbon coating in particular has proven effective: when a conductive carbon layer is applied to the surface of silicon particles, the resulting Si@C composite structure can simultaneously improve electronic conductivity, provide a mechanically compliant buffer against volume expansion, promote the formation of a more stable SEI film, and prevent direct contact between silicon and the electrolyte, thereby improving cycling performance. It should be noted, however, that the benefits of surface coating are strongly dependent on the specific coating material and its properties; not all coating approaches yield improved conductivity or structural stability, and some may introduce additional interfacial resistance or fail to accommodate silicon’s large volumetric changes. Carbon coating is the most widely adopted approach for silicon anodes owing to its intrinsic conductivity, chemical stability, and processability. In reference [23], artificial graphite-coated nano-silicon powder is obtained by spray drying, then vacuum heat-treated at 800 °C for 3 h, and finally carbonized in asphalt under a nitrogen atmosphere for 2 h at 900 °C to obtain a nano-silicon-enhanced graphite negative electrode material. In reference [24], a silicon/carbon/carbon (Si/C/C) composite material is successfully prepared by a two-step coating method. Currently, the process for realizing the nano-silicon-enhanced graphite negative electrode material is difficult, resulting in high manufacturing costs. Therefore, it has not been applied in practice. Although Betraye Company in China has successfully developed a nano-silicon-enhanced graphite negative electrode material and has achieved batch production and sales to Panasonic for making batteries, it has obvious problems of low production efficiency and high manufacturing costs in its current production process.

The mechanical grinding method mainly refers to the processing of materials through mechanical mixing. This method is easy to operate and control, and can obtain the required composite particles. It can support the production of composite materials. Reference [25] has conducted earlier research on this topic, using the mechanical ball milling method, which could handle two materials and prepare nano-silicon-enhanced graphite materials with different atomic ratios. Reference [26] has added a carbon source and then has used a high-temperature carbonization treatment method, which could obtain the required composite materials. In this case, the material’s cycle performance is significantly improved. However, in the nano-silicon-enhanced graphite materials compounded in this way, the carbon matrix on the outer layer would be damaged by the silicon’s volume expansion during the cycle process, and the stability is not good. Reference [27] has treated the mesophase carbon microspheres, making the silane material cover its surface. Reference [28] has obtained the nano-silicon-enhanced graphite materials through the method of high-temperature pyrolysis, by pyrolyzing the mixture of silicon and graphite at 1100 °C. Reference [29] has treated poly methyl methacrylate, added nano-silicon, thus forming a core–shell structure, and then processed it by the electrospinning method, finally obtaining the required composite materials.

Nano-silicon-enhanced graphite anode materials possess higher initial Coulombic efficiency and better rate performance. However, they still present certain inherent defects, including severe volume expansion and unsatisfactory cycling performance, which limit their application in diverse scenarios. Reference [30] has dissolved nano-active substances composed of natural graphite, elemental silicon and elemental tin, as well as resin-based organic carbon sources, in organic solvents. Reference [31] mixed nano-silicon with carbon materials, such as graphite, and further blended the mixture with crushed pitch, carbon nanotubes and hard carbon.

According to the principles of silicon nanocrystallization, the preparation methods of nano-silicon materials are mainly divided into chemical methods and physical methods. Physical methods primarily include mechanical ball milling and ion implantation, while chemical methods mainly cover precipitation and electrolysis. Chemical vapor deposition and mechanical ball milling are the most widely adopted approaches for nano-silicon preparation at present [32]. Nevertheless, both methods have inherent drawbacks. Mechanical ball milling mostly adopts dry grinding, which easily introduces impurities and reduces material purity. It also produces products with uneven particle size distribution and fails to achieve an ultra-fine limiting particle size. Although the nano-silicon prepared by chemical vapor deposition has a fine particle size and a relatively uniform particle size distribution, the preparation conditions are strict, and the process is complex.

Given the various limitations of the above methods for preparing nano-silicon, this paper adopts the segmented wet mechanical grinding method to achieve the nanosizing of silicon particles. This method has the characteristics of low limit particle size, very uniform particle size distribution, low energy consumption, high grinding efficiency, less pollution, and the retention of the properties of the ground particles. By optimizing the wet grinding process for silicon nanosizing and improving the equipment performance, the multi-stage grinding is changed to “coarse grinding + fine grinding”, which improves the grinding efficiency. The main research content of this paper is as follows: selecting low-cost silicon raw materials; studying the effects of solvents, dispersants, and grinding media on the wet grinding process for silicon nanocrystallization; researching the process of directly adding carbon sources to the wet grinding system to coat the nano-silicon, and using artificial graphite for the composite; and, finally, preparing nano-silicon-enhanced graphite negative electrode materials and studying their electrochemical properties.

## 2. Materials and Methods

### 2.1. Experimental Chemicals

The reagents required for this study are detailed in Table 1.

### 2.2. Experimental Instruments and Equipment

The main equipment used by the research institute is detailed in Table 2.

### 2.3. Sample Preparation for Material Characterization

#### 2.3.1. Particle Size Analysis

Aliquots of the nano-silicon dispersion are withdrawn directly from the sand mill at 30 min intervals without dilution and immediately transferred to the laser diffraction analyzer for measurement. Each sample is dispersed in isopropanol as the carrier medium, and the refractive indices of silicon and isopropanol are used for Mie-theory fitting. Three replicate measurements are performed per aliquot, and the D_50_, D_90_, and D_97_ values are recorded.

#### 2.3.2. Elemental Purity Analysis by ICP

Prior to ICP analysis, each silicon raw material is digested as follows: approximately 50 mg of silicon powder is weighed into a polytetrafluoroethylene (PTFE) vessel, to which 5 mL of hydrofluoric acid and 2 mL of nitric acid are added. The sealed vessel is heated at 180 °C for 2 h in a microwave digestion system until complete dissolution is achieved. The resulting solution is diluted to 50 mL with ultrapure water. Multi-element standard solutions at concentrations of 0.01, 0.05, 0.1, 0.5, and 1.0 mg/L are prepared from certified single-element stock solutions to construct the calibration curves. Blank digestions are performed in parallel to correct for background contamination. The concentrations of Fe, Cr, Ni, Zn, Ca, Mg, K, Na, P, and B are quantified simultaneously.

#### 2.3.3. Tap Density Measurement

Approximately 10 g of SMG powder is placed into a 25 mL graduated cylinder without compaction. The cylinder is then mounted on the tap density analyzer and subjected to 500 taps at a fixed drop height and frequency in accordance with ASTM B527. The final volume is read after the tapping sequence is complete, and the tap density is calculated as mass divided by final volume.

#### 2.3.4. XRD

All powder samples are ground gently in an agate mortar to break up soft agglomerates, then back-loaded into standard sample holders to minimize preferred orientation. XRD patterns are collected on a PANalytical X’Pert Pro diffractometer using Cu Kα radiation over a 2θ range of 10–80° at a scanning rate of 2°/min. Phase identification is performed by comparison with the JCPDS reference database, and the Scherrer equation is applied to the reflection of graphite and the reflection of silicon to estimate crystallite sizes.

#### 2.3.5. SEM

SMG composite powders are dispersed ultrasonically in anhydrous ethanol for 5 min, and a small drop of the suspension is deposited onto a silicon wafer substrate and allowed to dry in air. Prior to imaging, the sample surface is sputter-coated with a 5 nm gold layer to enhance conductivity and reduce charging artifacts. SEM images are acquired on a Hitachi SU8220 field-emission instrument at an accelerating voltage of 5 kV. Energy-dispersive X-ray spectroscopy (EDS) mapping is simultaneously performed to confirm the elemental distribution of Si, C, and O across the composite.

#### 2.3.6. X-Ray Photoelectron Spectroscopy (XPS)

XPS is performed using a monochromatic Al Kα X-ray source on an ESCALAB 250Xi spectrometer (Thermofisher, Waltham, MA, USA). Survey spectra are acquired at a pass energy of 100 eV over the binding energy range 0–1100 eV, and high-resolution Si 2p is collected at a pass energy of 20 eV with a step size of 0.05 eV. The binding energy scale is calibrated against the adventitious C 1s peak at 284.8 eV. Peak fitting and quantification are carried out using Shirley background subtraction followed by Voigt profile deconvolution in CasaXPS 2.3.14. XPS is conducted on (i) raw nano-silicon before grinding and (ii) nano-silicon after wet grinding in each of the four candidate solvents.

### 2.4. Electrochemical Performance Testing Equipment and Methods

#### 2.4.1. Cell Assembly and Configuration

All electrochemical measurements are performed using CR2032 coin-type half-cells assembled in an argon-filled glove box with O_2_ and H_2_O levels maintained below 0.1 ppm. The working electrode is prepared by mixing the SMG composite active material, conductive carbon black, and polyvinylidene fluoride (PVDF) binder in a mass ratio of 8:1:1 in the solvent N-methyl-2-pyrrolidone (NMP). The resulting slurry is cast onto copper foil using a doctor blade coater and dried at 80 °C for 12 h in a vacuum oven. The dried electrode sheets are roll-pressed and punched into discs of 12 mm diameter. Lithium metal foil served as the counter and reference electrode, a polyethylene microporous membrane is used as the separator, and 1 M LiPF_6_ dissolved in ethylene carbonate/dimethyl carbonate is used as the electrolyte. All cells are assembled in a two-electrode configuration.

#### 2.4.2. Galvanostatic Charge–Discharge Cycling

Galvanostatic cycling is conducted on a LAND battery testing system at 25 °C. For cycling performance evaluation, cells are cycled at a constant current density of 1C for 50 cycles within a voltage window of 0.001–1.5 V. For rate performance evaluation, cells are subjected sequentially to 5 cycles each at current densities of 0.1C, 0.2C, 0.5C, 1C, and 2C, followed by a return to 0.1C, all within the same voltage window of 0.001–1.5 V. The first-cycle Coulombic efficiency is calculated as the ratio of the first delithiation capacity to the first lithiation capacity.

#### 2.4.3. Cyclic Voltammetry (CV)

CV measurements are performed on an electrochemical workstation at 25 °C. The potential window is set from 0.001 V to 3.0 V at a scan rate of 0.1 mV/s. The first three consecutive cycles are recorded to assess SEI film formation, electrode activation behavior, and the reversibility of lithium insertion/extraction reactions in the SMG composite anode.

#### 2.4.4. Electrochemical Impedance Spectroscopy (EIS)

EIS measurements are performed on the CHI 660E electrochemical workstation at 25 °C after the cells had been cycled at 0.2C for 50 cycles. Spectra are collected over a frequency range of 100 kHz to 10 MHz with a sinusoidal AC perturbation amplitude of 5 mV applied at open-circuit potential. The obtained Nyquist plots are fitted using the equivalent circuit model, where Re denotes the internal resistance of the circuit, R_SEI_ denotes the SEI film resistance, R_ct_ denotes the charge-transfer resistance, and W denotes the Warburg diffusion element.

## 3. Results and Discussion

A pure silicon anode has the characteristic of high theoretical capacity, which is more than 10 times that of graphite. However, the expansion effect during alloying leads to capacity loss, and the disadvantage of poor conductivity is also very obvious. Theoretically, the nanosizing of silicon can help alleviate and inhibit the volume expansion caused by alloying of silicon anode material during the full-electric process. Through nanosizing, it helps silicon particles release stress through volume expansion, thereby destroying their microstructure and reducing the loss of lithium ions, promoting further improvement in the cycling performance.

### 3.1. Selection of Silicon Raw Materials

**Figure 1:**
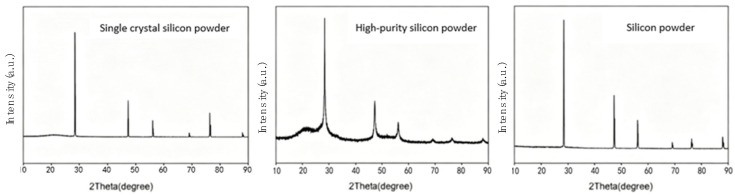
XRD spectra of single-crystal silicon, high-purity silicon, and metallic silicon.

This article selects common high-purity silicon powder, metallic silicon, and single-crystal silicon cutting materials available on the market as silicon raw materials. The main focus is on analyzing the content of impurity elements and selecting the appropriate silicon as the raw material by comprehensively considering cost factors. Table 3 presents the detection data of 24 impurity elements (Fe, Cr, Ni, Zn, Ca, Mg, K, Na, P, B) in the above three silicon raw materials through ICP elemental analysis. Figure 1 shows the XRD spectra of the three silicon raw materials. From the spectra, it can be seen that the peak shapes of all three are relatively sharp and the crystallinity is good. Thus, it can be observed that the purity of the three raw materials is basically similar. The single-crystal silicon raw material is the cutting material for photovoltaic single-crystal silicon, and its cost is relatively lower, but it has a higher content of metal impurities and is prone to oxidation, so it is not suitable as a raw material. Compared to high-purity silicon powder, metallic silicon powder has a lower cost, so metallic silicon powder is chosen as the silicon raw material.

### 3.2. Selection of Solvents

**Figure 2:**
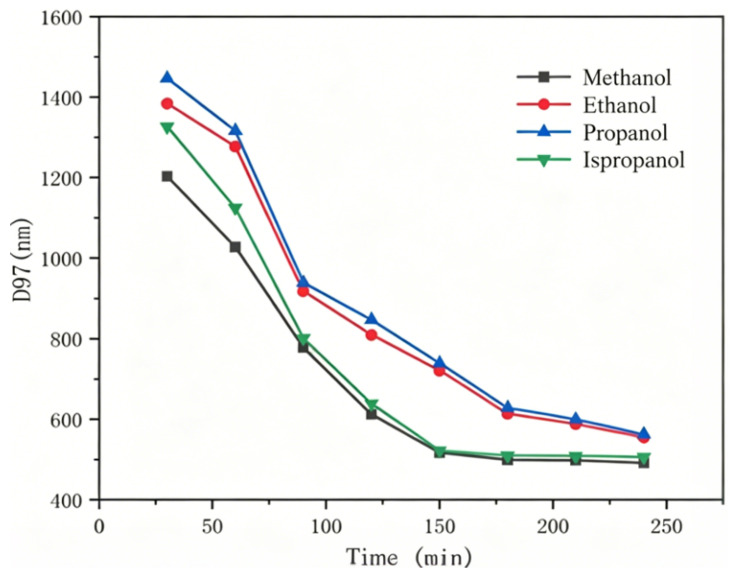
Comparison chart of grinding effects with different solvents.

When grinding the silicon raw material to the nanometer level through wet grinding, an appropriate solvent needs to be added. The solvent not only carries the nanoscale silicon but also has the effect of preventing the aggregation and oxidation of the nanoscale silicon. This is to prevent the surface of the silicon from being oxidized during nanoscale scaling, and at the same time, it plays a role in dispersion to prevent the aggregation of the nanoscale silicon. Although non-polar n-hexane can effectively prevent the oxidation of the nanoscale silicon, its strong adsorption property affects the grinding effect. Non-polar deionized water cannot protect the nanoscale silicon from oxidation. Therefore, this paper selects methanol, ethanol, propanol, and isopropanol as grinding solvents. Using a sand mill under the same equipment parameters, ball-to-solvent ratio, and solid content, the silicon raw material with a particle size of 5 μm is ground. Samples are taken every 30 min to test the particle size of the silicon after grinding. The specific data are shown in Figure 2. From the figure, it can be seen that methanol has the best grinding effect, followed by isopropanol slightly.

### 3.3. Selection of Dispersants

**Figure 3:**
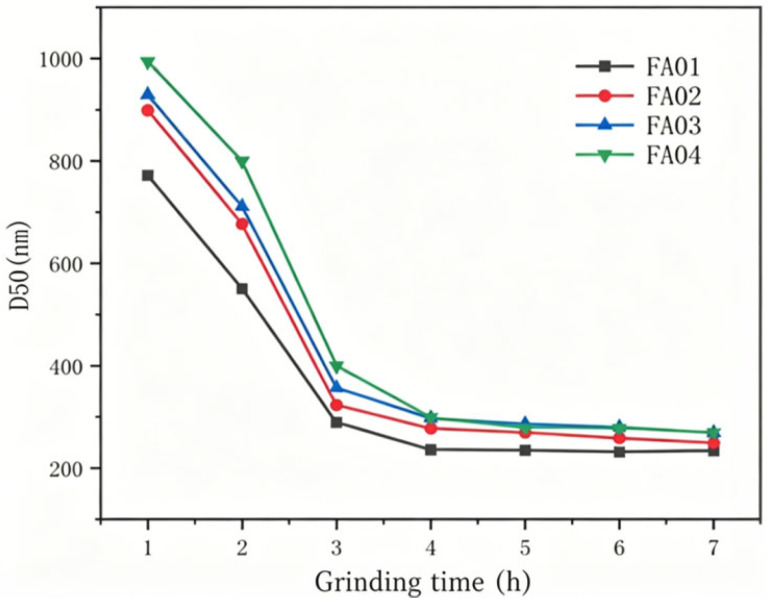
The influence of dispersants on the grinding effect.

Commonly used dispersing agents for grinding silica nanoparticles include carboxylic acids, amines, polyacrylic acids, and phosphate esters. To select the more effective dispersing agent for grinding and dispersing nano-silica, four types of dispersing agents—FA01 (carboxylic acids), FA02 (amines), FA03 (polyacrylic acids), and FA04 (phosphate esters)—are selected for comparative experiments. The metal silicon powder, different dispersing agents, and isopropanol are mixed in a ratio of 1:1:8 by mass and then fed into a high-speed disperser. The high-speed disperser is operated at a speed of 1500 rpm for 10 min of dispersion time. After the liquid is well mixed, it is placed into a sanding equipment through the feeding port, using 0.5 mm grinding media, with a ball-to-media ratio of 1:2.5, a solid content of 8%, and a grinding speed of 15 m/s. Once the instrument is started, the metal silicon powder is added, and the grinding process lasts for 1 h. Samples are taken to test the particle size distribution of silicon. This operation is repeated every 1 h. Specific data are shown in Figure 3. From the figure, it can be seen that when FA01 is used as the dispersing agent, the grinding effect is the best.

### 3.4. The Influence of Grinding Process on Nano-Silicon

To reduce the size of silicon powder from micrometer-sized to nanometer-sized, it is necessary to decrease its particle size by two orders of magnitude. In the case of wet grinding, for each reduction of one order of magnitude, the size of the grinding medium must be reduced accordingly to provide sufficient force to the small particles in the grinding system, achieving the best grinding effect and efficiency.

Using a high-speed disperser, metal silicon powder, FA01 and isopropanol are mixed in a 1:1:8 ratio evenly. This mixture is rotated at 1500 rpm for 10 min. Then, the liquid is added to the sand mill, and grinding media of 1.0 mm, 0.5 mm, 0.2 mm, and 0.1 mm are selected, with a ball-to-powder ratio of 1:2.5 and a solid content of 5%. A grinding speed of 15 m/s is used. The prepared metal silicon powder sample is added and ground for 1 h. Samples are taken to test the particle size distribution of silicon. This operation is repeated every 1 h.

**Figure 4:**
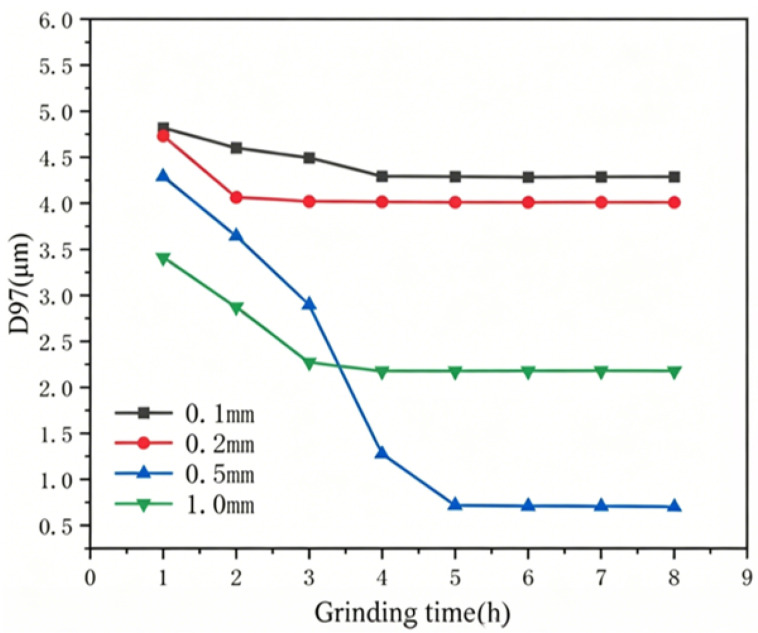
Comparison of the variations in silicon particle size D_50_ value with grinding time when using 0.1 mm, 0.2 mm, 0.5 mm, and 1.0 mm grinding media, respectively.

Figure 4 shows the comparison of the changes in the particle size distribution of silicon with the grinding time when using grinding media of 0.1 mm, 0.2 mm, 0.5 mm, and 1.0 mm, respectively. From the figure, it can be seen that, for the 5 μm silicon raw material, when the grinding medium is 1.0 mm, the D_97_ value decreases rapidly within the first 3 h and the grinding efficiency is high, but from the 4th hour, the D_97_ value basically remains at 2.1 μm and no longer changes; when the grinding medium is 0.5 mm, the D_97_ value decreases rapidly within the first 5 h and the grinding efficiency is high, and the D_97_ value has reached 0.7 μm after grinding for 5 h; and when the grinding medium is 0.1 mm and 0.2 mm, the particle size changes very little after grinding for 8 h and does not reach the expected effect.

**Figure 5:**
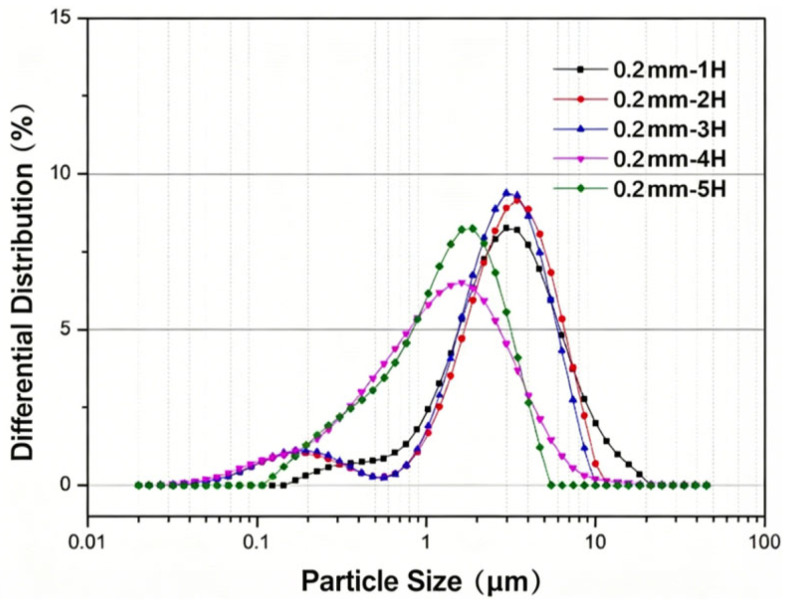
Comparison chart of particle size distribution curve of silicon with time when using 0.2 mm grinding medium.

Figure 5 shows the curve of the particle size distribution of the silicon raw material over time when it is ground using a 0.2 mm grinding medium. From the figure, it can be seen that, in the first 3 h, the majority of the particles are still large. However, a small bulge appeared at 0.3–0.5 μm. This is because, before the grinding began, the large silicon particles had many fine particles adhering to their surfaces. These fine particles gradually detached from the surface of the large particles as the grinding continued. As the grinding process progressed to the 4th hour, the distribution of the particle sizes of the large particles changed very little because the grinding medium was too small and did not match the particle size of the material to be ground, and was unable to provide sufficient energy to break the large particles.

**Figure 6:**
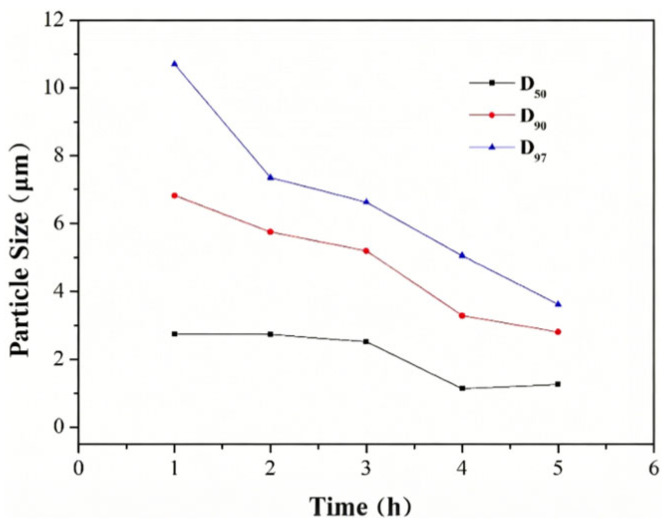
Trend chart of D_50_, D_90_, and D_97_ values when using 0.2 mm grinding medium.

Figure 6 shows that, when using a 0.2 mm grinding medium, the values of the silicon’s particle size distribution D_50_, D_90_ and D_97_ all change to some extent over time. It is obvious that, in the first 3 h, the values of D_50_ and D_90_ slightly decreased as the grinding time progressed, but even after 5 h of grinding, the total change is not significant. Meanwhile, the value of D_97_ hardly changed with the increase in grinding time, which further confirms that the 0.2 mm grinding medium has an extremely poor grinding effect and very low grinding efficiency for 5 μm silicon raw materials. The 0.2 mm grinding medium is not compatible with the grinding of micrometer-sized silicon raw materials.

**Figure 7:**
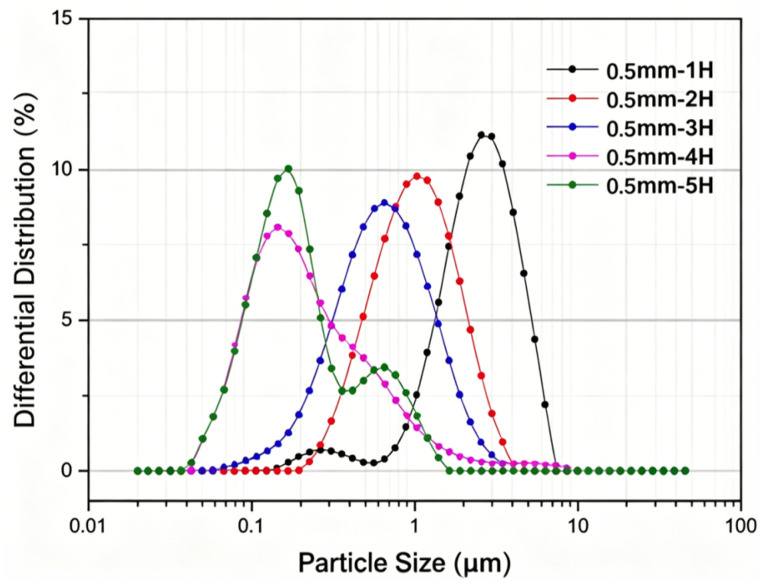
Comparison chart of particle size distribution curve of silicon after grinding with 0.5 mm grinding medium for different times.

**Figure 8:**
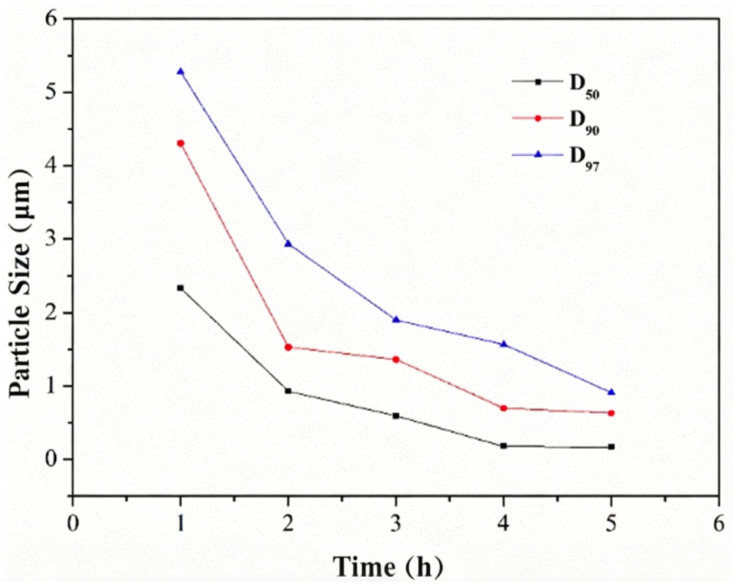
Trend chart of D_50_, D_90_, and D_97_ values when using 0.5 mm grinding medium.

Figure 7 shows the comparison of the particle size distribution curves of the silicon raw material after being ground for different periods using a 0.5 mm grinding medium. From the figure, it can be directly observed that, as the grinding progresses, the overall particle size distribution curve of the silicon raw material keeps shifting to the left, indicating that the overall particle size distribution range of the silicon raw material is continuously shrinking, and the silicon raw material is being broken down into smaller particles. After 3 h of grinding, the D_97_ of the silicon raw material dispersion solution decreased from 5.109 μm to 2.626 μm. Compared to the grinding effect with a 0.2 mm grinding medium, the grinding effect and efficiency of the 0.5 mm grinding medium on the selected 5 μm silicon raw material are very obvious. However, as the grinding continues, the progress of the particle size distribution curve of the silicon raw material shifting to the left gradually slows down. The distribution of small particles no longer increases, and the decrease in the distribution of large particles becomes extremely slow. This is because, as the material to be ground continues to be broken down, the originally matching grinding medium becomes too large and becomes incompatible. If the grinding medium is too large, the gaps produced by its accumulation are also relatively large, and for the already relatively smaller ground silicon raw material particles, the grinding effect becomes worse. Figure 8 shows the curves of D_50_, D_90_, and D_97_ of the silicon raw material dispersion solution with respect to the grinding time when using a 0.5 mm grinding medium. It is obvious that, in the 4 h–5 h period, the D_50_ value and D_90_ value did not change significantly. This means that, as the grinding progresses, the number of small particles increases and the number of large particles decreases, and the efficiency of grinding the large particles also decreases.

The matching of grinding media is a crucial factor in the grinding practice of sand mills. Based on the grinding theory of sand mills and the above experimental results, if the micrometer-sized particles are gradually ground down to the sub-micrometer level, media with a specification greater than 0.5 mm should be used; if the particles are at the sub-micrometer or nanometer level, media with a specification of 0.2 mm or below should be used. The energy required for grinding or dispersion depends on the difference between the initial particle size and the target particle size of the material to be ground. By matching different grinding media according to different differences, the grinding efficiency and effect can be greatly improved. In addition, by setting a reasonable grinding linear speed and adjusting appropriate parameters such as filling rate and ball ratio, better grinding effect and efficiency can be achieved.

**Figure 9:**
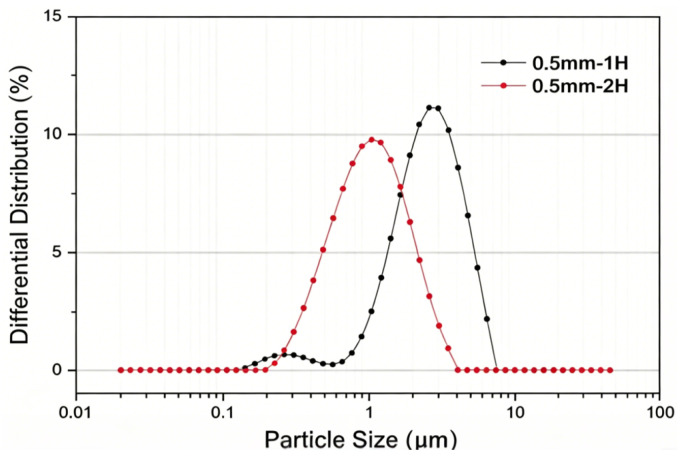
Comparison of particle size distribution curve after rough grinding every 1 h.

**Figure 10:**
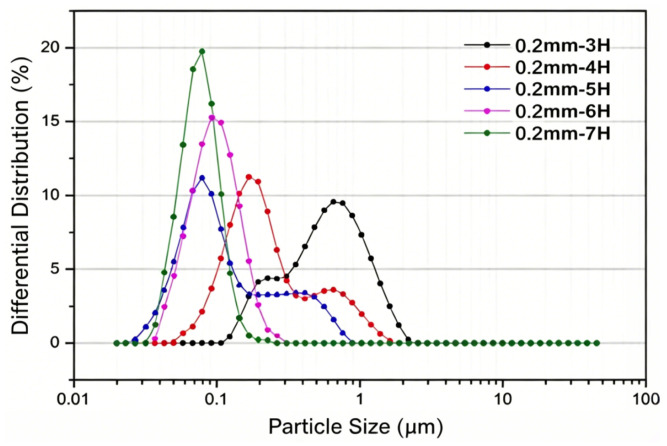
Comparison of particle size distribution curve after fine grinding every 1 h.

In conclusion, in order to improve the efficiency and effectiveness of grinding, it would be ideal to adopt the method of “coarse grinding + fine grinding” for segmented grinding treatment. During the coarse grinding process, when the material is relatively large, coarse grinding media should be used, which can quickly grind the material to a certain fineness. Then, for the material after coarse grinding, smaller and matching grinding media should be used for further grinding. Based on the above experimental results, in the coarse grinding stage, 0.5 mm grinding media are used for 2 h, and the starting particle size of silicon raw materials of 5 μm is ground to the sub-micron level; in the fine grinding stage, 0.2 mm grinding media are used for grinding for 5 h. Figure 9 and Figure 10 are the comparison diagrams of the particle size distribution curves of the silicon raw materials after each 1 h of grinding in the coarse and fine grinding stages. From the figures, it can be seen that, after fine grinding, all particle sizes are less than 200 nm, which can achieve the expected goal.

### 3.5. Preparation of SMG Materials

**Figure 11:**
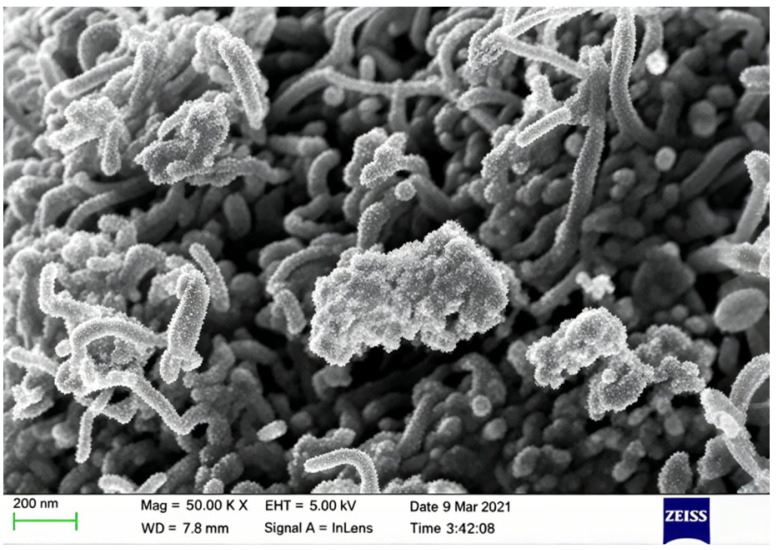
The SEM of MWCNTs.

First, the multi-walled carbon nanotubes are air-sieved to a particle size of approximately 5 μm. Then, a multi-walled carbon nanotube dispersion solution is prepared according to the method for preparing nano-silicon. In a ratio of 1:1 by solid content, a certain amount of nano-silicon dispersion solution and multi-walled carbon nanotube dispersion solution is taken, respectively, and they are sequentially fed into the sand mill through the feeding port for mechanical grinding and mixing. The ratio of nano-silicon dispersion solution to multi-walled carbon nanotube dispersion solution is 1:1 according to their solid contents. A grinding medium of 0.2 mm is selected, the filling rate is set to 90%, and the grinding linear speed of the sand mill is set to 15 m/s. After grinding for 30 min, a certain amount of artificial graphite is slowly added to the grinding slurry, and the grinding is continued for another 30 min. Then, the slurry is released and dried at 350 °C to obtain SMG. By adjusting the graphite dosage, different samples are fabricated. Among them, the SMG sample with an 8% silicon content is not prepared, as the resulting slurry exhibited excessively high viscosity. Figure 11 shows the SEM image of the multi-walled carbon nanotubes.

### 3.6. XPS Analysis of Nano-Silicon Surface Oxidation During Wet Grinding

To quantitatively assess the extent of grinding-induced oxidation, XPS characterization of Si 2p is conducted on nano-silicon samples before grinding and after wet grinding in each of the four candidate solvents. An additional sample subjected to IPA wet grinding followed by the 350 °C Ar annealing step is also analyzed to evaluate whether post-grinding heat treatment partially reverses oxidation.

**Figure 12:**
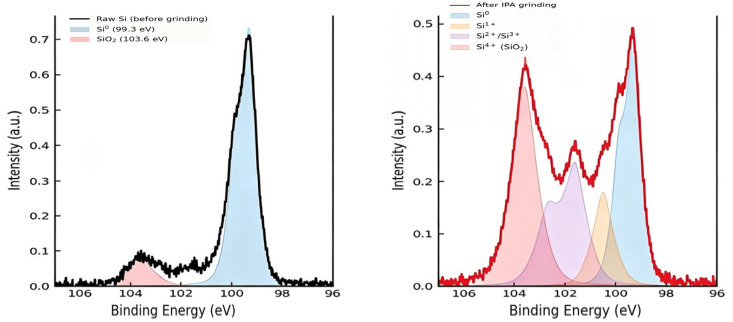
The Si 2p XPS spectrum of nano-silicon.

Figure 12 presents the Si 2p high-resolution XPS spectra of raw nano-Si and nano-Si after IPA wet grinding. In the raw nano-Si spectrum, the dominant peak at 99.3 eV corresponds to elemental silicon, with only a minor SiO_2_ component at 103.6 eV accounting for 7.3 at.% of total silicon—consistent with a thin native oxide layer inherently present on commercial nano-silicon powder. After IPA wet grinding, the spectrum is markedly altered: the Si^0^ peak intensity decreases substantially, and four oxidation-state components are clearly resolved: Si^1+^, Si^2+^/Si^3+^, and Si^4+^/SiO_2_. In total, the oxidized silicon fraction increases from 7.3 at.% to 41.8 at.% after IPA grinding, confirming that significant surface oxidation does occur during the wet grinding process.

### 3.7. The Composition and Morphology of SMG Material

**Figure 13:**
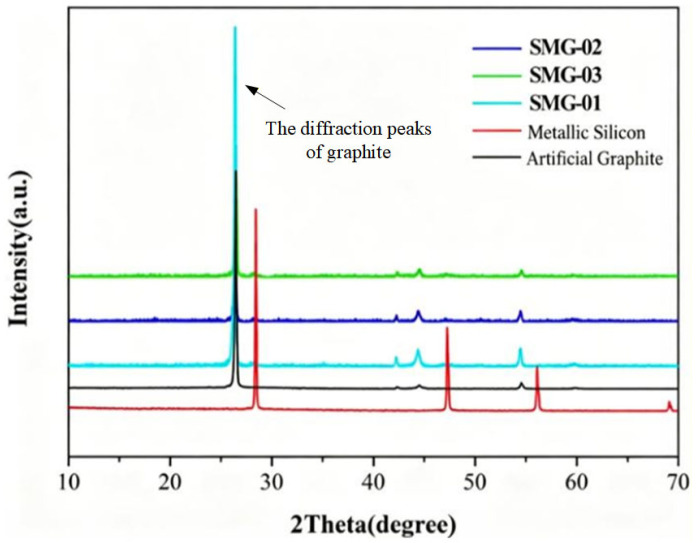
The XRD patterns of SMG silicon–MWCNT composites and raw materials.

The XRD characterization is conducted on the raw materials of metallic silicon, artificial graphite, and the prepared materials, as shown in Figure 13. By comparing and analyzing the XRD spectra of SMG samples with different silicon contents and the XRD spectra of the raw materials, the following can be concluded: Firstly, the diffraction peaks of silicon in the XRD spectrum of the SMG negative electrode material still exist, and the peak shape is as sharp as that of silicon in the raw material, indicating that the crystal form of silicon has not changed after it is mechanically ground and coated with carbon nanotubes to form a complex-structured SMG. Secondly, no obvious impurity peaks are observed in the XRD spectrum of the SMG negative electrode material, indicating that no new phases are generated during the material preparation process. The several raw materials only underwent physical changes during the preparation process and combined to form a new spatial structure. In all SMG samples, the characteristic diffraction peak of graphite remains sharp and symmetric at approximately 2θ = 26.5°, with no significant shift in peak position or broadening of the full width at half maximum compared to the raw graphite. This indicates that the long-range ordered layered structure of graphite is well preserved after high-intensity sand milling. The absence of any pronounced peak broadening or emergence of amorphous halos in the XRD patterns confirms that the milling process did not induce measurable structural disordering or amorphization of the graphite phase. From this, it can be concluded that the sand-milling mixing process adopted in this experiment has caused minimal disturbance to the crystal structure of artificial graphite during the preparation of the SMG negative electrode material. The layered ordered structure of the graphite matrix remains intact after high-intensity mechanical mixing, which is also consistent with the SEM morphology observation results.

**Figure 14:**
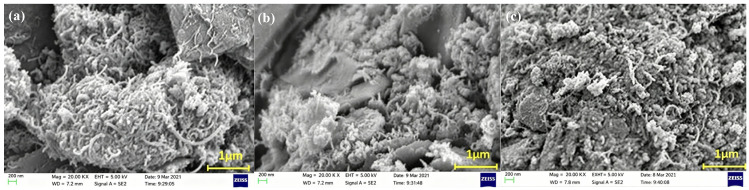
(**a**) SMG-01 SEM image. (**b**) SMG-02 SEM image. (**c**) SMG-03 SEM image.

Figure 14 shows the SEM images of SMG with different silicon contents. From the figure, it can be clearly seen that carbon nanotubes are uniformly coated on the surface of nano-silicon, and the formed coating is evenly distributed on the graphite matrix. This indicates that, during the high-intensity mechanical grinding process, multi-walled carbon nanotubes cross-wound and formed a complex spatial network structure, which well encapsulated the nano-silicon. Due to the presence of this encapsulation layer, the gradual consumption of active Li^+^ by the silicon was effectively avoided, thereby preventing the significant loss of battery capacity. Moreover, the encapsulation layer formed by multi-walled carbon nanotubes on nano-silicon also has a certain inhibitory and buffering effect on the volume expansion of nano-silicon during the charge and discharge cycles.

### 3.8. Analysis of the Cyclic Voltammogram of SMG

**Figure 15:**
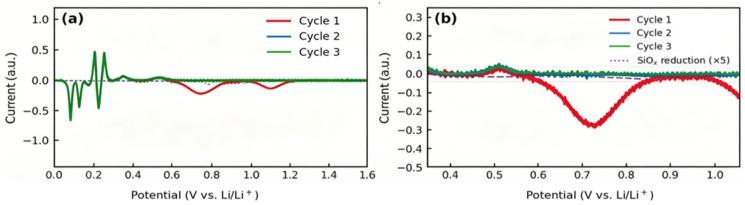
(**a**) SMG-01 CV. (**b**) Enlarged view of SMG-01 in the 0.35–1.05 V range.

Figure 15 shows CV analysis of SMG composite materials with SiO_x_ reduction. Figure 15a shows SMG-01 CV, and Figure 15b is an enlarged view of SMG-01 in the 0.35–1.05 V range. As can be seen from Figure 15a, in SMG-01, silicon constitutes only 2 wt% of the composite. The XPS-determined surface oxidation of 41.8 at.% Si refers to the silicon atomic fraction at the particle surface, not bulk composition. For 22 nm Scherrer-diameter Si nanoparticles with a 2–4 nm oxide shell, the volumetric fraction of SiO_x_ is approximately 40–60% of the total silicon particle volume. However, given that silicon itself is only 2 wt% of the composite electrode, the SiO_x_ mass fraction in the total electrode is at most 1 wt% of the electrode. The theoretical capacity associated with SiO_2_ reduction corresponds to approximately 1790 mAh/g for pure SiO_2_. At 1 wt% SiO_2_ in the electrode, the maximum expected SiO_2_ reduction capacity is thus only 17.9 mAh/g—representing merely 4.8% of the total first-cycle discharge capacity. This capacity is spread over a broad voltage window, yielding a very small differential current that is below the signal-to-noise ratio of standard CV measurements.

In Figure 15b, the dotted curve shows the theoretical SiO_x_ reduction signal calculated for SMG-01 at its actual predicted amplitude. This predicted signal is smaller than the measurement noise floor, explaining definitively why no distinct SiO_x_ reduction peaks are observed in the experimental CV curves.

To provide a direct experimental control demonstrating that the electrochemical signals in the CV curves originate from silicon rather than from the graphite matrix or from grinding-induced graphite defects, a graphite-only reference electrode (Gr-ref) is prepared using an identical wet grinding procedure in an isopropanol medium, without any nano-silicon. The Gr-ref electrode thus undergoes the same mechanical and chemical treatment as the graphite component in the SMG composites, enabling unambiguous attribution of additional electrochemical features to silicon.

**Figure 16:**
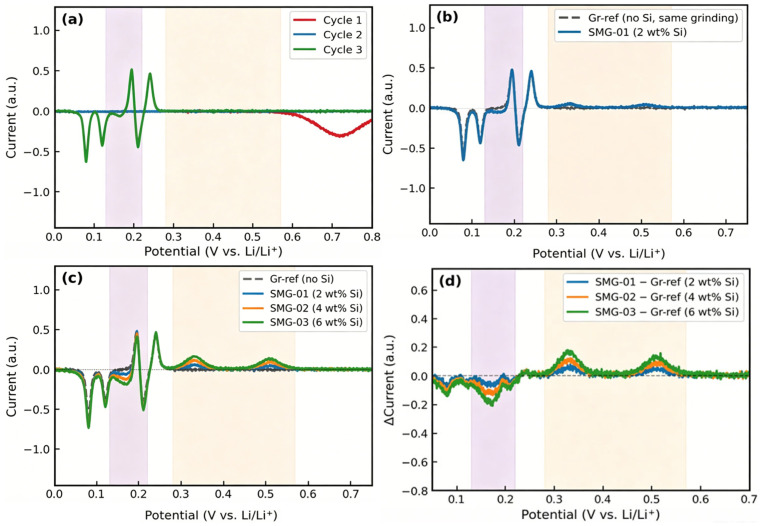
(**a**) Gr-ref CV cycles 1-3. (**b**) Cycle-3 overlay of Gr-ref vs. SMG-01. (**c**) Cycle-3 comparison across Gr-ref and all three SMG samples. (**d**) Difference curves ΔCV = SMG − Gr-ref.

Figure 16 presents the comparative CV analysis. The purple and yellow background colors respectively indicate whether cathode peaks and anode peaks occur at 0.17 V, 0.33 V and 0.51 V. Figure 16a shows the CV cycles 1–3 of Gr-ref: only the three well-known graphite staging peaks are observed. Crucially, no features are detected at 0.17 V, 0.33 V, or 0.51 V in the Gr-ref electrode across all three cycles. These three positions are explicitly marked with “no signal” annotations, confirming that the grinding process itself does not generate any additional electrochemical signatures in the graphite electrode. Figure 16b directly overlays the cycle-3 CVs of Gr-ref and SMG-01: the SMG-01 curve exhibits three additional features—a cathodic shoulder at 0.17 V and anodic peaks at 0.33 and 0.51 V. These three peaks correspond precisely to the characteristic electrochemical signatures of amorphous silicon. Figure 16c extends the comparison to all three SMG samples: the intensities of all three Si-specific features increase monotonically from SMG-01 to SMG-03, tracking the silicon loading, while the graphite staging peaks remain unchanged in position and amplitude. Figure 16d shows the difference curves ΔCV = SMG − Gr-ref, which directly isolate the pure silicon electrochemical contribution. The ΔCV signals resolve three clean peaks at 0.17, 0.33, and 0.51 V with amplitudes proportional to silicon loading, providing the most direct possible evidence that these signals arise from silicon alloying reactions and not from graphite or any other component.

### 3.9. The First Charge–Discharge Analysis of SMG

**Figure 17:**
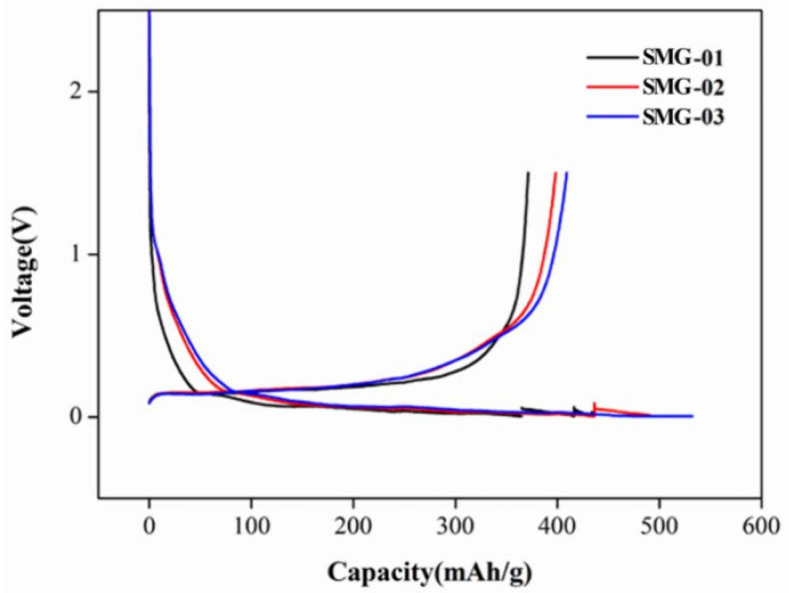
First charge and discharge curve of SMG01-03.

Figure 17 shows the first charge–discharge curves of three groups of SMG materials with different silicon contents. As can be seen from the figure, the charge–discharge curves of all three groups of SMG samples all show a clear lithium-alloying–dealloying voltage plateau. The charge–discharge curves of all three groups of SMG samples have a low and long-lasting discharge plateau near 0 volts to 0.2 volts, corresponding to the lithium-alloying process. Moreover, the curves are very flat in the range of 0.2 volts to 0.5 volts, corresponding to the dealloying process. There is no graphite discharge plateau within 1.0 volts to 1.2 volts, mainly because silicon has more alloying–dealloying channels than graphite, and the charge–discharge process of the SMG materials is mainly the alloying and dealloying of lithium ions in silicon. The first discharge capacities of the three groups of SMG samples are 373.7 mAh/g, 395.9 mAh/g, and 415.6 mAh/g, respectively, and the first lithiumization capacities are 443.2 mAh/g, 498.4 mAh/g, and 541.7 mAh/g, respectively. The first Coulomb efficiencies of the three groups of SMG are 84.32%, 79.43%, and 76.72%, respectively, with significant irreversible capacity losses. To further enhance the Coulomb efficiency, pre-lithiation technology is currently one of the most effective methods. The specific method is to directly mix the micron-sized lithium powder with the negative electrode slurry or press it onto the surface of the electrode sheet. Under the activation of a small amount of moisture, the lithium powder spontaneously intercalates into the graphite-dominant material, compensating for the consumption of the SEI. However, the first charge efficiency of the three groups of SMG negative electrodes is significantly higher than that obtained when pure silicon is used as the negative electrode. Thus, it can be concluded that covering silicon with carbon nanotubes can more effectively reduce the irreversible capacity loss caused by the volume expansion of silicon alloying. A more detailed cross-sample comparison reveals clear trends driven by silicon content. The irreversible capacity increases systematically from SMG-01 to SMG-02 and SMG-03. This monotonic increase directly reflects the growing proportion of nano-silicon, which possesses a higher specific surface area and therefore generates a larger absolute quantity of SEI film during the first lithiation. For SMG-01 with only 2 wt% Si, the dominant electrochemical contribution comes from the graphite matrix, which inherently exhibits lower irreversible capacity and more stable SEI formation behavior, resulting in the highest initial Coulombic efficiency (ICE) of 84.32% among the three samples. In contrast, SMG-03 exhibits the largest first lithiation capacity but the lowest ICE, owing to the intensified silicon alloying reaction, which generates both a thicker initial SEI layer and greater volumetric strain in the electrode. Despite the progressively lower ICE from SMG-01 to SMG-03, all three samples still outperform reported pure nano-silicon anodes, confirming that MWCNT encapsulation combined with graphite compounding effectively moderates irreversible lithium consumption.

To quantitatively attribute the measured capacity between graphite and nano-silicon contributions, the Gr-ref electrode is also subjected to GCD testing under identical conditions. The Gr-ref first discharge capacity is 361.0 mAh/g, with a first-cycle ICE of 84.1%. This value serves as the empirical graphite baseline capacity under the actual processing conditions, because wet grinding in isopropanol introduces a small degree of graphite surface defects that modestly reduce its practical capacity.

**Figure 18:**
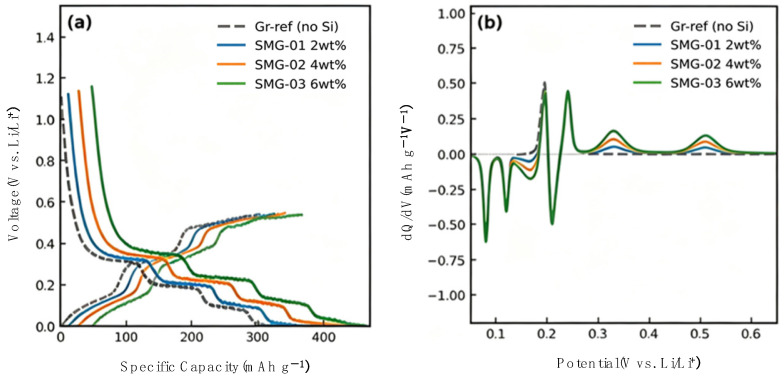
(**a**) First-cycle discharge curves of Gr-ref and SMG-01/02/03. (**b**) ICA comparison.

Figure 18 presents the GCD control results. Panel (a) overlays the first discharge curves of Gr-ref and SMG-01/02/03: the Gr-ref curve shows the characteristic graphite voltage profile with staging plateaus at 0.08–0.21 V, while the SMG curves show an additional capacity region extending beyond 361 mAh/g, directly confirming silicon’s capacity contribution. The ICA analysis in Panel (b) resolves this more clearly: the Gr-ref shows only graphite staging peaks, while the SMG samples exhibit additional ICA features at 0.17 V and 0.33/0.51 V that are absent in Gr-ref and scale proportionally with silicon loading.

### 3.10. Cyclic Performance Analysis of SMG

**Figure 19:**
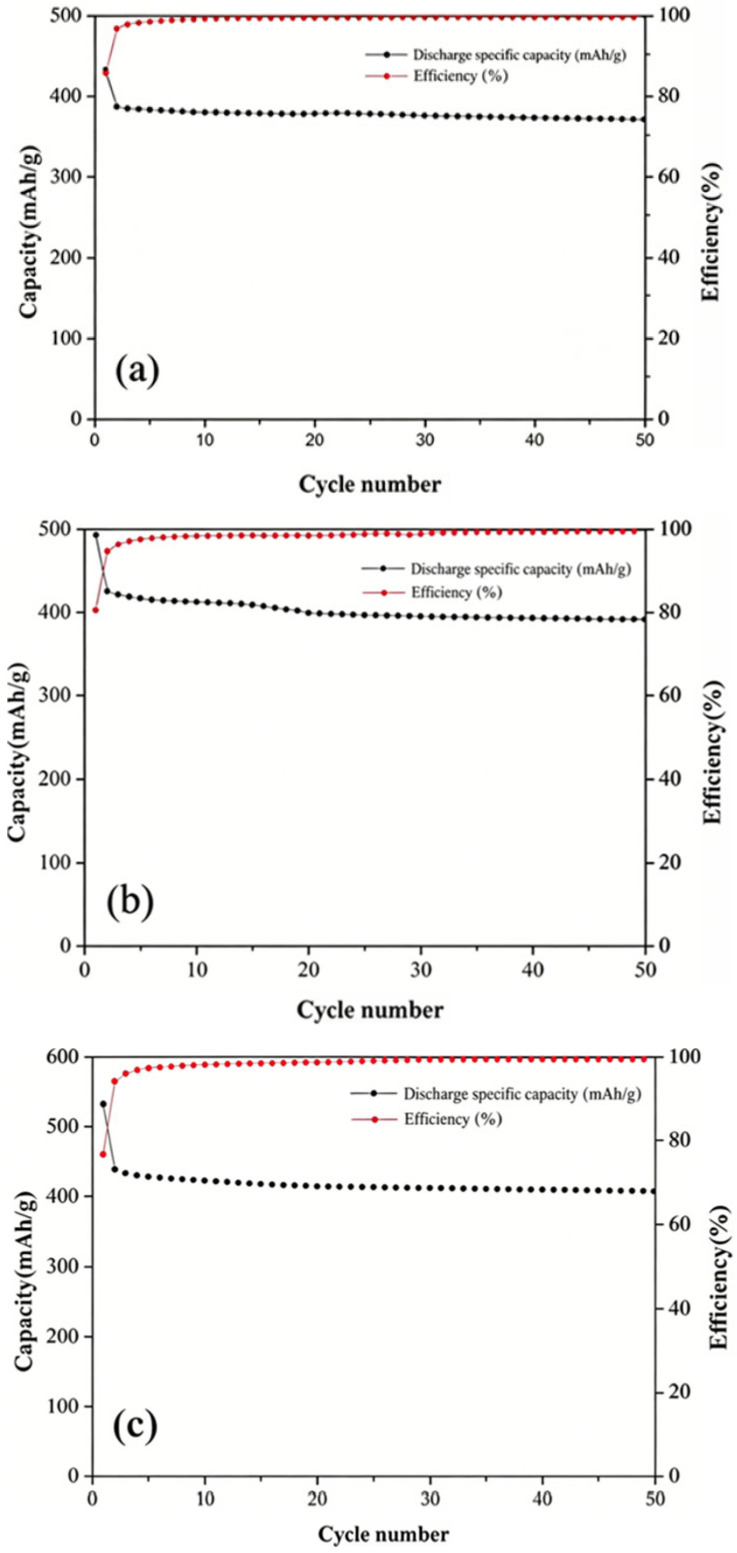
The cyclic performance of the three groups of SMG materials. (**a**) SMG-01 cycle performance chart. (**b**) SMG-02 cycle performance chart. (**c**) SMG-03 cycle performance chart.

Figure 19 shows the 1C cycling performance of three sets of SMG negative electrode materials at room temperature (25 °C). As can be seen from the figure, by the second sweep of cycling, the specific capacity of the three samples has significantly decreased, mainly due to the formation of the SEI film, which causes the loss of active Li+ in the material and thus leads to irreversible loss of discharge capacity. However, the charging and discharging efficiency from the third sweep onwards has exceeded 90%, indicating that the network structure of carbon nanotubes has a better effect on the surface coating of silicon, effectively preventing silicon from directly contacting the electrolyte, promoting the formation and stability of the SEI film, and improving the cycling performance of the material. With the increase in the number of cycles, the discharge specific capacity of the material slightly decreases, but the change is very small. After 50 cycles, the capacities of the three sets of SMG negative electrode materials are 359.2 mAh/g, 364.7 mAh/g, and 388.2 mAh/g, respectively, which are 96.12%, 92.12%, and 93.41% of the capacity after the second cycle. It is also found that the stable discharge specific capacity of the three sets of SMG negative electrode materials is positively correlated with the silicon content in the material. In addition, the graphite matrix exists in the system, which increases the conductivity of the system. Comparing the three samples more closely, SMG-01 achieves the highest capacity retention of 96.12% after 50 cycles, followed by SMG-03 and SMG-02. Although one might expect a monotonic decline in retention with increasing silicon content due to accumulating volumetric strain, SMG-02 shows slightly poorer retention than SMG-03. This may be attributed to a non-uniform distribution of nano-silicon within the SMG-02 at the 4 wt% loading: at this intermediate silicon content, silicon particles may be insufficiently dispersed within the MWCNT network, leaving localized silicon-rich zones that undergo more severe, heterogeneous volume expansion than in SMG-03, where a higher silicon content may paradoxically encourage more thorough MWCNT entanglement and encapsulation during the grinding process.

### 3.11. Analysis of the Rate Performance of SMG

**Figure 20:**
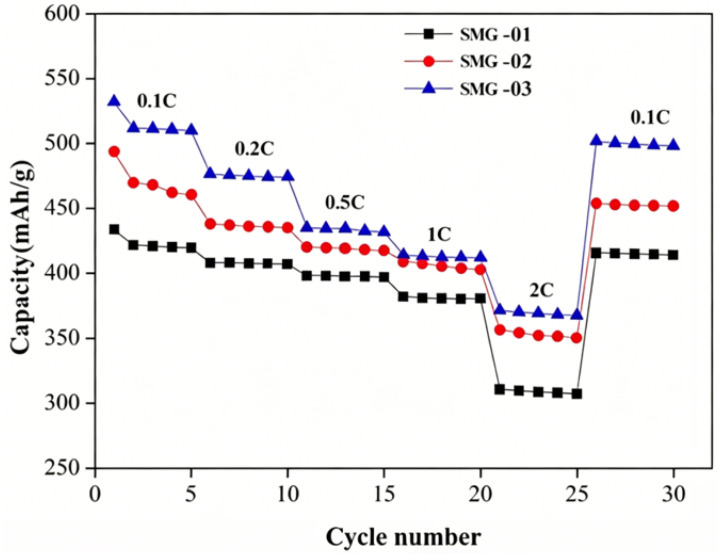
SMG01-03 voltage-rate performance curve.

Figure 20 shows the rate performance curves of three sets of SMG anode materials. It is easy to observe from the figure that, during the first sweep of the 0.1C discharge cycle, all three groups of samples experienced significant capacity loss. This is also due to the formation of the SEI film, resulting in some loss of lithium ions. At a current density of 2C, the discharge specific capacity of the three SMG samples with different silicon contents remained relatively stable, indicating that the materials have good rate performance under high current density. When the current density is restored from 2C to 0.1C, the final capacity retention rates of SMG01-03 relative to the capacity of the second cycle are 98.71%, 96.16%, and 97.36%, respectively. The marginally lower capacity recovery rates of SMG-02 and SMG-03 are consistent with greater mechanical degradation at higher silicon loadings under repeated high-rate cycling, where increased volumetric strain around nano-silicon particles progressively impairs electrode structural reversibility.

### 3.12. Electrochemical AC Impedance Spectroscopy Analysis of SMG

**Figure 21:**
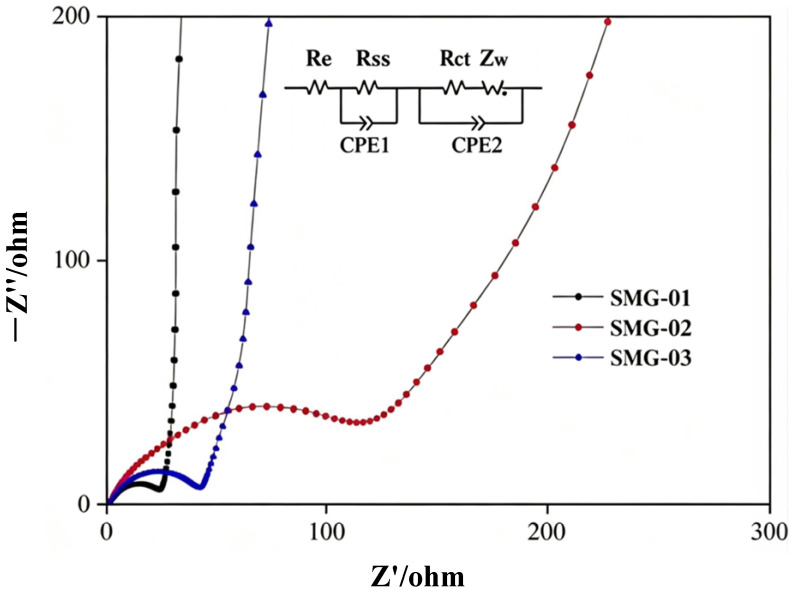
SMG01-03 AC impedance spectroscopy and equivalent circuit.

Figure 21 shows the EIS spectra and equivalent circuit diagrams of three groups of SMG anode materials after being made into button cells and cycling at 0.2C for 50 cycles at room temperature. In the equivalent circuit, Re represents the internal resistance of the battery, R_SEI_ is the resistance value in the SEI film, and R_ct_ is the conductive resistance existing at each interface. From the figure, it can be seen that the shapes of the AC impedance spectra of the three groups of SMG anode materials are consistent. The spectra in the low-frequency range are similar to a straight line, while in the medium- and high-frequency ranges, the main feature is a semi-circular arc that protrudes upwards. The former corresponds to the ion diffusion behavior, and the latter describes the conduction behavior of lithium ions in the SEI film. A quantitative comparison of impedance parameters from equivalent circuit fitting clarifies electrochemical disparities between SMG-01, SMG-02 and SMG-03. Internal resistance Re, originating from electrolyte and contact resistance, should remain comparable for all three samples under identical cell assembly, with trivial deviations induced by subtle differences in electrode thickness and porosity stemming from varied silicon fractions. Charge-transfer resistance R_ct_ offers more valuable information, quantifying the kinetic barrier to lithium desolvation and interfacial electron transport at electrode–electrolyte boundaries. After 50 cycles, SMG-01 with low silicon content retains a stable electrode framework and delivers lower R_ct_ than SMG-03, signifying a less-damaged, better-maintained electrode–electrolyte interface. SMG-02 displays abnormal, disordered impedance curves.

**Figure 22:**
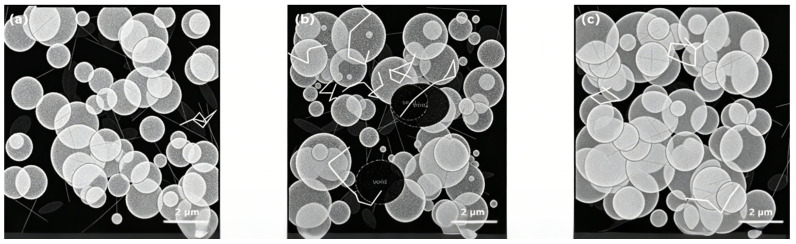
SEM morphology comparison diagram after the cycle. (**a**) SMG-01 after 50 cycles. (**b**) SMG-02 after 50 cycles. (**c**) SMG-03 after 50 cycles.

By comparing the AC impedance spectra of the three groups of SMG anode materials, it is found that the spectrum of SMG-02 is significantly different from the other two samples and has no regularity. According to the SEM simulation images of the electrode cross-section of the three groups of samples after 50 cycles, as shown in Figure 22, this is due to the severe pulverization of SMG-02 particles, the expansion of multiple cracks, and the occurrence of local peeling cavities.

**Figure 23:**
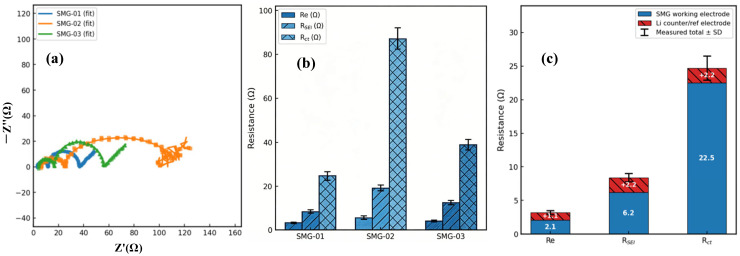
(**a**) The Nyquist plots of the experimental data and the fitting curves. (**b**) Fitted impedance parameters R_e_, R_SEI_, and R_ct_ for all three samples. (**c**) Impedance attribution for SMG-01.

Figure 23 shows the repeatability of the EIS measurement results and the impedance attribution. First, to demonstrate measurement reproducibility, Figure 23a shows the Nyquist plots with the three replicate data points and the mean fit curve for each sample, providing a visual representation of the inter-cell reproducibility. Figure 23b presents the fitted impedance parameters as bar charts with error bars representing one standard deviation across the three replicates. The standard deviations are ±0.3–0.8 Ω for Re, ±0.5–1.2 Ω for R_SEI_, and ±1.5–4.8 Ω for R_ct_. The relative ordering SMG-01 < SMG-03 for both R_SEI_ and R_ct_ is consistent across all nine cells, and none of the confidence intervals overlap between SMG-01 and SMG-03, confirming that the observed trends are statistically reproducible and not attributable to cell-to-cell variability.

Second, all EIS measurements are performed in two-electrode CR2032 coin half-cells, in which lithium metal foil serves simultaneously as a counter electrode and a reference electrode. In this configuration, the measured impedance is the sum of contributions from the SMG working electrode and the lithium metal electrode. The lithium metal electrode contributes its own charge-transfer resistance, ohmic contact resistance, and a passivation layer impedance that evolves during cycling. Consequently, the absolute values of the fitted Re, R_SEI_, and R_ct_ parameters reported below cannot be unambiguously attributed solely to the SMG working electrode material; they carry a systematic uncertainty from the lithium electrode contribution. Figure 23c illustrates this uncertainty by decomposing the measured SMG-01 impedance into the estimated working electrode contribution and the estimated lithium counter/reference electrode contribution. The lithium electrode contribution to Re and R_SEI_ is estimated at 1.1 and 2.2 Ω, respectively; the contribution to R_ct_ is 2.2 Ω.

### 3.13. Role of MWCNTs: Comparison with Si@Graphite (SG) Control Samples

**Figure 24:**
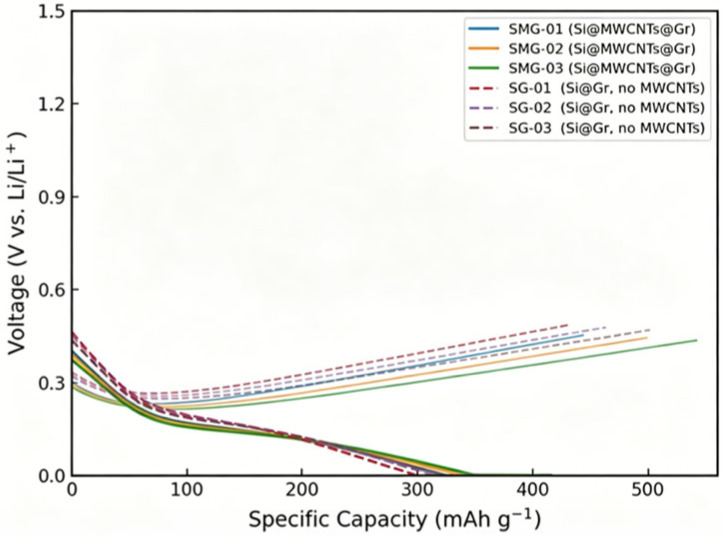
First galvanostatic charge–discharge profiles of SMG-01/02/03 and SG-01/02/03 at 0.05 C.

**Figure 25:**
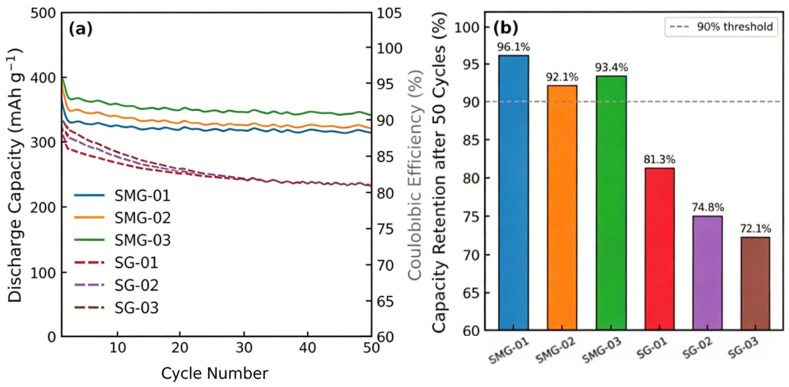
(**a**) Discharge capacity vs. cycle number at 1 C for SMG-01/02/03 and SG-01/02/03. (**b**) Bar chart comparing capacity retention after 50 cycles at 1 C.

**Figure 26:**
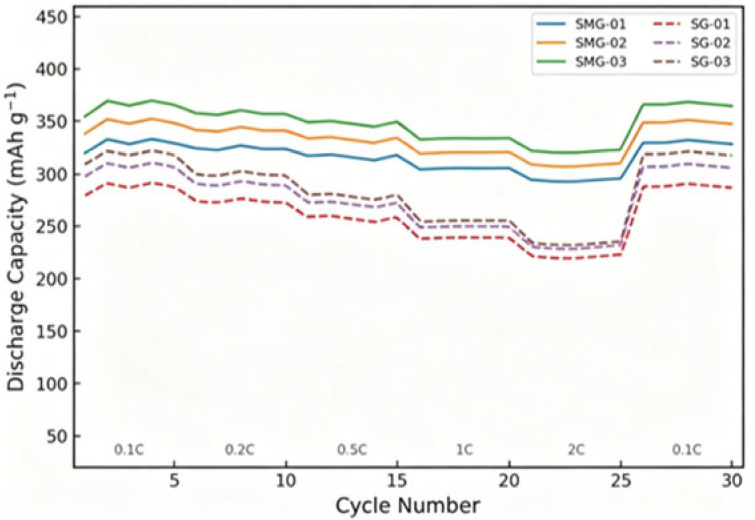
Rate performance comparison of SMG-01/02/03 and SG-01/02/03.

To unambiguously isolate the electrochemical contribution of MWCNTs in the SMG, three control samples without MWCNTs are prepared following an identical procedure but omitting the MWCNT dispersion step. These control samples, designated SG-01, SG-02, and SG-03, retain the same silicon-to-graphite mass ratio as the corresponding SMG samples. Their electrochemical performance is compared side-by-side with SMG-01/02/03 in Figure 24, Figure 25 and Figure 26 below.

Figure 24 presents the first charge–discharge profiles of all six samples. The SG control samples exhibit markedly lower ICE than their SMG counterparts: SG-01 achieves only 75.6% ICE, SG-02 achieves 68.2%, and SG-03 achieves 65.4%. The significantly lower ICE of SG samples is attributed to the absence of the MWCNT encapsulation layer, which in SMG samples serves as a conformal protective coating that limits the direct exposure of nano-silicon to the electrolyte, thereby suppressing the formation of excessive SEI film during the first lithiation cycle. Without MWCNTs, nano-silicon particles in the SG are directly embedded in the graphite matrix with only weak van der Waals contacts, leading to poorer interfacial passivation and larger irreversible lithium consumption. Additionally, the first discharge capacities of SG samples are substantially lower than those of SMG samples, despite having identical silicon contents.

Figure 25 shows the 1 C cycling stability over 50 cycles and the corresponding capacity retention comparison. The MWCNT-containing SMG samples demonstrate decisively superior cycling stability compared with SG controls. After 50 cycles, SMG-01 retains 96.12% of its second-cycle capacity, while SG-01 retains only 81.3% under identical conditions. The difference is even more pronounced for higher silicon loadings: SG-02 retains only 74.8%, and SG-03 retains only 72.1%. The pronounced capacity fade of SG samples is a direct consequence of the unconstrained volume expansion of nano-silicon during repeated lithiation–delithiation cycling. Without the MWCNT buffer network, the large volumetric strain fragments the silicon particles and disrupts the electrical contact between silicon and the graphite current carrier, causing progressive electrode pulverization.

Figure 26 compares the rate capability of SMG and SG samples across current densities from 0.1 C to 2 C. SMG samples exhibit substantially superior rate performance at all current densities. At 2 C, SMG-01 retains 89.1% of its 0.1 C capacity, while SG-01 retains only 76.8%. The advantage of MWCNTs in high-rate operation stems from two synergistic effects: (i) the highly conductive MWCNT network dramatically reduces the charge-transfer resistance and facilitates rapid electron delivery to silicon particles, and (ii) the shortened lithium-ion diffusion pathways within the MWCNT-encapsulated nano-silicon domains allow fast solid-state Li^+^ transport even at high current densities. SG samples, lacking this conductive scaffolding, rely entirely on the graphite matrix for electron transport to silicon, which is kinetically insufficient at high rates and results in significant polarization and capacity loss. When the current density is restored to 0.1 C after high-rate cycling, SMG samples recover 98.71%, 96.16%, and 97.36% of their second-cycle capacity for SMG-01/02/03, respectively, while SG samples recover only 88.4%, 83.7%, and 82.2% for SG-01/02/03, confirming superior structural reversibility in the MWCNT-containing samples.

### 3.14. Comparison of Electrochemical Performance with Si@graphene@graphite (SGG) Materials

#### 3.14.1. Preparation of SGG Materials

In a ratio of solid content 1:1, a certain amount of nano-silicon dispersion solution and graphene dispersion solution is taken, respectively, and they are sequentially fed into the sand mill through the feeding port for mechanical grinding and mixing. The ratio of nano-silicon dispersion solution to graphene dispersion solution is 1:1 according to their solid contents. A grinding medium of 0.2 mm is selected, the filling rate is set to 90%, and the grinding linear speed of the sand mill is set to 15 m/s. After grinding for 30 min, a certain amount of artificial graphite is slowly added to the grinding slurry, and grinding is continued for 30 min. Then, the slurry is released and dried at 350 °C to obtain SGG. By adjusting the different amounts of graphite added, the silicon content of the SGG negative electrode material is controlled to be 2%, 4%, 6%, and 8%, respectively, and they are recorded as SGG-01, SGG-02, SGG03, and SGG-04.

#### 3.14.2. Analysis of SGG CV Curve

**Figure 27:**
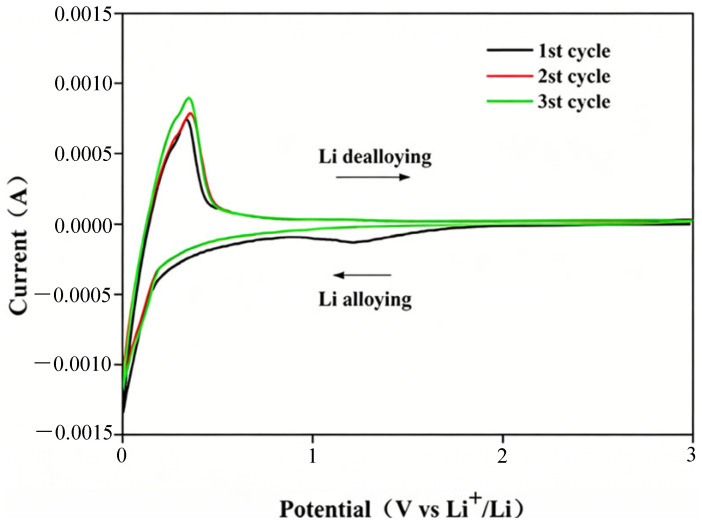
The CV curve graph of the SGG material.

The lithium insertion and extraction behaviors of SGG negative electrodes with different silicon contents are characterized within the voltage range of 0.001 volts to 3.0 volts. The results are shown in Figure 27. It can be seen that the CV curves of SGG in the first sweep and the following two sweeps are different. During the cycling lithium insertion segment of the first cycle, an irreversible reduction peak with a wide but low intensity can be found in the range of 1.0 volts to 1.3 volts, and it did not appear in the subsequent two sweeps of cycling. Thus, it can be concluded that an irreversible SEI film is formed during the first cycle of cycling, and the formed SEI film is stable without repeated growth and rupture. Therefore, this irreversible peak did not appear in the subsequent CV cycling curves. As the cycling continued, a sharp and strong cathode peak with a peak shape around 0.02 volts is observed, mainly due to lithium being inserted into silicon, resulting in amorphous Li_x_Si. In addition, at the 0.3 volts to 0.4 volts position in the anode part, a relatively obvious oxidation peak is present, indicating that lithium had been discharged from the lithium–silicon alloy phase. This is known as lithium extraction. When the cycling reached the second and third sweeps, the intensity of the anode peak increased, but the position did not change significantly. The intensity of the cathode peak at 0.02 volts also slightly increased, indicating that the first three sweeps of cycling are the gradual activation process of the electrode.

#### 3.14.3. SGG’s First Charge–Discharge Analysis

**Figure 28:**
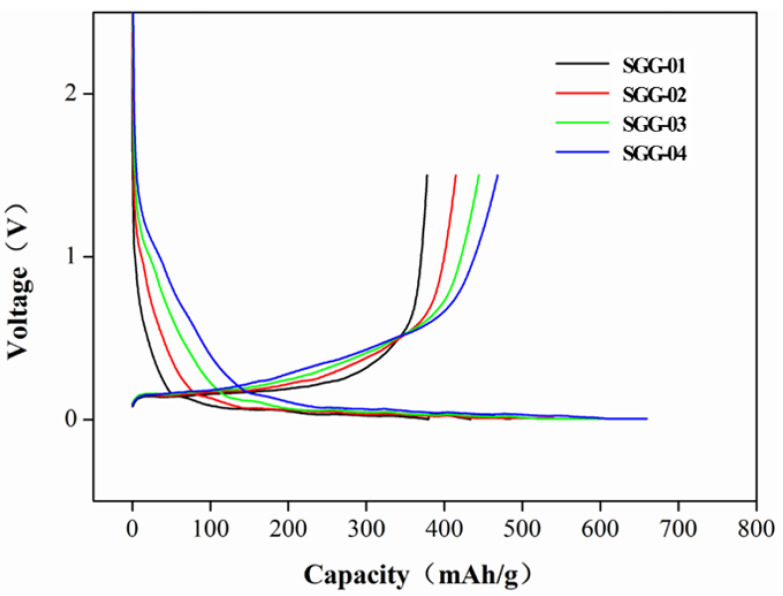
The first charge–discharge curves of the four groups of SGG materials.

Figure 28 shows the first charge–discharge curves of four groups of SGG material samples with different silicon contents. In all four groups of SGG samples, a clear lithium-alloying–dealloying voltage platform can be observed. There is a relatively low discharge plateau in the range of 0 volts to 0.2 volts in the charge–discharge curves of all four groups of SGG samples, which lasts for a relatively long time and corresponds to the complete process of silicon alloying. Within the range of 0.2 volts to 0.5 volts, the curves are relatively flat, indicating the dealloying process. The typical discharge plateau of graphite does not appear. The possible reason is that the lithium-ion alloying and dealloying channels of silicon are more than those of graphite, and the charge–discharge process of the SGG negative electrode material is mainly dominated by the alloying and dealloying of lithium ions in silicon. The initial lithium storage capacity of the four samples is 486.8 mAh/g, 542.7 mAh/g, 602.9 mAh/g, and 659.8 mAh/g, respectively, and the initial dealloying capacity is 379.3 mAh/g, 417.1 mAh/g, 446.5 mAh/g, and 469.6 mAh/g, respectively. The initial Coulombic efficiency of the four SGG negative electrode materials is 77.92%, 76.86%, 74.06%, and 71.17%, respectively, and the irreversible capacity is above 20%. This is mainly because when the material is charging and discharging, due to the alloying of silicon, there is a volume expansion, which forms volume stress in the electrode sheet, causing the electrode sheet to fall off or powderize. However, the first charge efficiency of the four SGG negative electrode materials is significantly higher than that of pure silicon used as the negative electrode, indicating that after graphene coating of silicon, the volume expansion during silicon alloying can be effectively reduced, and the capacity retention rate of the material can be improved.

#### 3.14.4. SGG Cycle Performance Analysis

**Figure 29:**
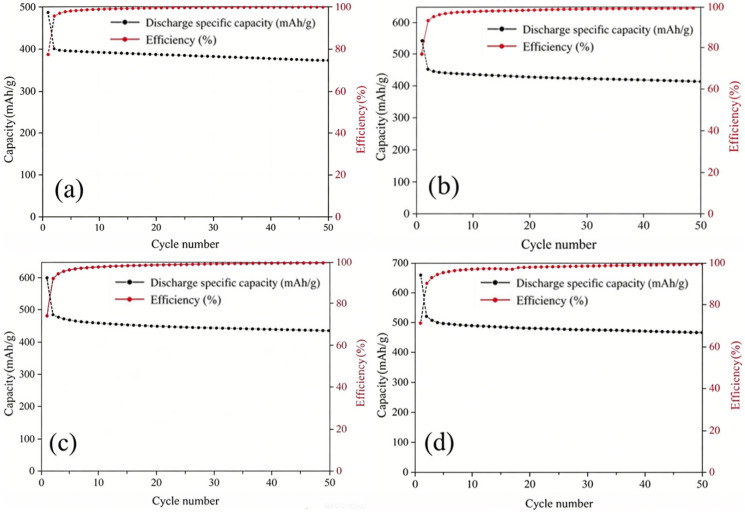
(**a**) SGG-01 cycle performance diagram. (**b**) SGG-02 cycle performance diagram. (**c**) SGG-03 cycle performance diagram. (**d**) SGG-04 cycle performance diagram.

Figure 29 shows the 1C cycling performance of four sets of SGG negative electrode materials at room temperature (25 °C). As can be seen from the figure, during the cycling of all samples, the specific capacity decreased significantly in the first and second sweeps, mainly due to the formation of the SEI film, which led to the loss of active Li+ and caused the irreversible loss of discharge capacity. However, the charging and discharging efficiency from the third sweep onwards is always above 90%, indicating that the graphene coating on the surface of silicon effectively prevented silicon from directly contacting the electrolyte, promoted the formation and stability of the SEI film, and improved the cycling performance of the material. With the increase in the number of cycles, the discharge specific capacity of the material slightly decreased, but the change is very small. After 50 cycles, the capacities of the four sets of SGG negative electrode materials are 353.4 mAh/g, 383.9 mAh/g, 402.8 mAh/g and 421.3 mAh/g, respectively, which are 93.17%, 92.04%, 90.21% and 89.71% of the capacity after the second cycle. In addition, the graphite matrix exists in the system, which not only increases the conductivity of the system, but more importantly, buffers the volume expansion of silicon during charging and discharging, helping to improve the stability of the cycling.

#### 3.14.5. SGG Rate Performance Analysis

**Figure 30:**
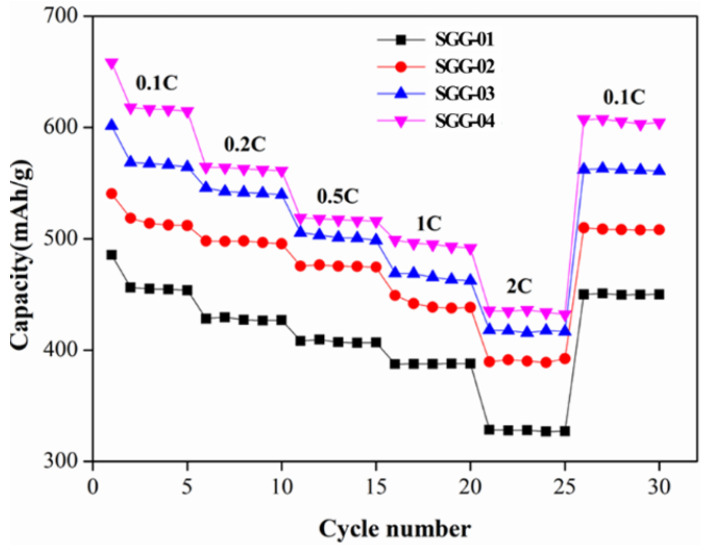
Performance curve of the magnification for SGG01-04.

As shown in Figure 30, the rate performance diagram of the SGG01-04 sample is presented. From the figure, it can be seen that when the current density is 2C, the capacities of the four silicon-containing samples remain stable. After the current density is restored from 2C to 0.1C, the final capacity retention rates of SGG01-04 are 92.70%, 94.00%, 93.23%, and 91.79%, respectively. This indicates that SGG has excellent rate performance. The SGG-02 sample and SGG-03 sample have higher capacity retention rates than the SGG01 sample. The main mechanism is that the increase in nano-silicon enhances the capacity retention rate of the material, while the addition of an appropriate amount of graphene increases the overall effect of encapsulation, thereby suppressing the volume expansion phenomenon caused by silicon alloying. In addition, the SGG-04 sample has the lowest capacity retention rate. This is mainly because excessive silicon is added, causing significant volume expansion of the electrode sheet, and the negative impact brought about by this exceeds the positive impact of the increase in silicon content on the capacity retention rate.

#### 3.14.6. SGG Electrochemical AC Impedance Spectroscopy Analysis

**Figure 31:**
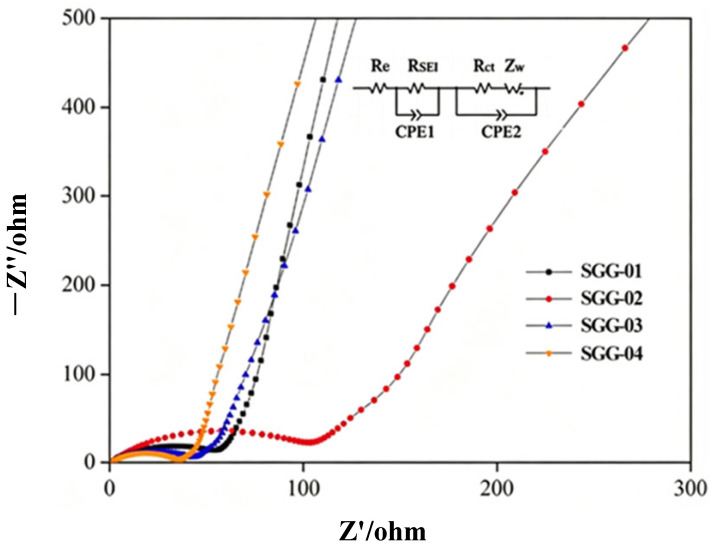
EIS diagrams and equivalent circuits of the four groups of SGG samples.

Figure 31 shows the equivalent circuit diagram and EIS spectrum of four groups of SGG anode materials after being made into button cells and undergoing 0.2C cycling for 50 cycles at room temperature. Here, R_e_ represents the internal resistance of the circuit, R_SEI_ is the resistance of the SEI film, and R_ct_ indicates the conductive resistance between different interfaces within the battery. It is easy to observe from the figure that the shapes of the EIS spectra of the four groups of SGG anode materials are consistent. In the low-frequency range, the corresponding spectra are all close to a straight line, while in the medium- and high-frequency range, they are an upwardly protruding semi-circular arc. The spectra in the low-frequency range correspond to the process of ion diffusion, and the spectra in the medium- and high-frequency range represent the transmission of lithium ions within the SEI film. Through the analysis of the obtained experimental results, it can be known that the EIS spectrum of SGG-04 is significantly different from the other three samples. This might be because, as the ratio of nano-silicon to graphite increases, the conductivity of silicon itself is poor, which lowers the conductivity of the material.

Based on the above experimental results, when the current density returned to 0.1C, the final capacity retention rate of the SGG01-04 sample reached over 90%, while that of the SMG01-03 sample reached over 95%. Therefore, the SMG anode material has better rate performance compared to the SGG anode material. The initial Coulomb efficiency of SGG01-04 is 77.92%, 76.86%, 74.06%, and 71.17%, while the initial Coulomb efficiency of SMG01-03 is 84.32%, 79.43%, and 76.72%. After cycling 50 times at a 1C current density, the capacity of SMG01-03 is 96.12%, 92.12%, and 93.41% of the capacity of the second cycle, respectively. Thus, the SMG anode material has better stability compared to the SGG anode material.

### 3.15. Limitations and Future Work

This study has achieved the simple preparation of carbon-coated nano-silicon materials and has verified their excellent electrochemical properties. However, there are still many shortcomings. The uniformity of the thickness of the carbon coating layer is difficult to precisely control. Local coating is weak, and the problem of cracking of the carbon layer cannot be avoided. The volume expansion suppression effect of the silicon-based material is limited in long-term cycling. At the same time, the preparation process is highly sensitive to parameters such as reaction temperature and heating rate. The experimental repeatability needs to be improved. Moreover, the batch production of the material is difficult, and problems such as particle agglomeration and coating failure are prone to occur during mass production. Additionally, no systematic exploration has been conducted on the material’s stability under extreme conditions such as high temperature and high current density.

Future work will optimize the preparation process parameters, combine modification methods to construct an ultra-thin, dense and highly elastic composite carbon-coated structure, and strengthen the structural stability. At the same time, quantitative characterization techniques will be introduced to precisely control the coating morphology and thickness, improving the experimental repeatability. Moreover, the optimization of the large-scale production process will be carried out, testing the material’s performance under extreme conditions, and exploring the material’s energy storage mechanism, further enhancing its cycle life and rate performance, and promoting its industrial application.

## 4. Conclusions

This paper investigates the influence of silicon raw material selection, solvent type, dispersant type, and grinding process on the wet grinding process for preparing nano-silicon, as well as the preparation process and electrochemical properties of the SMG material. The conclusions are as follows:

(1) This paper systematically investigates various types of components during the mechanical grinding process in the preparation of nano-silicon dispersion, as well as the effects of different grinding processes on the preparation of nano-silicon dispersion. Moreover, a stably dispersed nano-silicon dispersion liquid is successfully prepared. Using 5 μm metallic silicon powder as the raw material, isopropanol as the solvent, and FA01 (carboxylic acid type) as the dispersant, the “coarse grinding + fine grinding” two-stage grinding method is adopted. In the coarse grinding stage, the silicon raw material is ground with 0.5 mm grinding media for 2 h, and in the fine grinding stage, it is ground with 0.2 mm grinding media for 5 h. Eventually, a stable nano-silicon dispersion liquid is obtained.

(2) The first lithium ion discharge capacities of SMG01-03 are 373.7 mAh/g, 395.9 mAh/g and 415.6 mAh/g, respectively. The initial Coulombic efficiencies of SMG01-03 are 84.32%, 79.43%, and 76.72%, respectively. The higher the silicon content, the higher the specific capacity of the anode.

(3) After cycling 50 times at a current density of 1 C, the capacities of SMG01-03 are 96.12%, 92.12%, and 93.41% of the capacity after the second cycle. After silicon nanocrystallization and surface coating, it is indeed able to effectively alleviate the volume expansion effect of silicon during charging and discharging, and prevent the continuous growth and rupture of the SEI film caused by the direct contact of silicon with the electrolyte in the battery system, thereby ensuring that the negative electrode material has good cycling performance.

(4) The final capacity retention rate of the SMG01-03 samples has always reached over 95%. The carbon layer covering the nano-silicon particles can, to some extent, limit the volume changes during the silicon alloying process, which helps form a stable SEI film and enables the anode to better accommodate and release lithium ions.

## Figures and Tables

**Table 1:** Experimental reagents.

Experimental Chemicals	Specification/Model	Manufacturer
High-purity silicon	5 mm	Zhongsi High-Tech Co., Ltd., Luoyang, China.
Silicon metal	5 mm	Hoshine Silicon Industry Co., Ltd., Jiaxing, China.
Monocrystalline silicon	5–10 mm	LONGi Green Energy Technology Co., Ltd., Xian, China.
Anhydrous ethanol	AR	Yonghua Chemical Co., Ltd., Changshu, China.
Methanol	AR	PetroChina Company Limited, Beijing, China.
Propanol	AR	Jiangsu Yabang Chemical Co., Ltd., Lianyungang, China.
Isopropyl alcohol	AR	Aladdin Biochemical Technology Co., Ltd., Shanghai, China.
FA01 (Carboxylic Acid Dispersant)	AR	Sanzheng New Materials Co., Ltd., Tianjin China.
FA02 (Amino-based Dispersant)	AR	Tinci Materials Technology Co., Ltd., Guangzhou, China.
FA03 (Polyacrylic Acid-Type Dispersant)	AR	Zhejiang Kaidi New Materials Co., Ltd., Hangzhou, China.
FA04 (Phosphate Ester Dispersant)	AR	Jiangsu Feiya Chemical Industry Co., Ltd., Nantong, China.
Metal lithium sheet	Battery grade	Ganfeng Lithium Group Co., Ltd., Xinyu, China.
Copper foil	Battery grade	Jiangtong Copper Foil Technology Co., Ltd., Nanchang, China.
Snap-type battery module	Battery grade	BYD Company Limited, Shenzhen, China.
Conductive carbon black	Battery grade	Black Cat Carbon Black Co., Ltd., Jingdezhen, China.
N-Methyl pyrrolidone	Battery grade	Jiangsu Yatai Chemical Co., Ltd., Nantong, China.
Electrolyte	Battery grade	Guotai Huarong New Chemical Materials Co., Ltd., Zhangjiagang, China.
Diaphragm	Battery grade	Senior Technology Material Co., Ltd., Shenzhen, China.
Artificial graphite	KD-6	BTR New Material Group Co., Ltd., Shenzhen, China.
Carbon nanotubes	NGC01	Cnano Technology Limited, Beijing, China.
Graphene	Slurry SF01	Morsh Technology Co., Ltd., Ningbo, China.

**Table 2:** Experimental devices.

Instrument/Equipment Name	Model Number	Manufacturer
Numerical Control Ultrasonic Cleaner	KQ2200DB	Kunshan Shumei, Suzhou, China.
Vibrating machine	DAS200	Tai Jing Technology Co., Ltd., Suizhou, China.
Mechanical crusher	Customization	Shangshui Intelligent Technology, Shenzhen, China.
Air flow pulverizer	Customization	Shangshui Intelligent Technology, Shenzhen, China.
Grinding mill	10 L, Customization	Shangshui Intelligent Technology, Shenzhen, China.
Disc dispersion machine	HTS-250	Daming Chemical Machinery, Dongguan, China.
X-ray diffractometer (XRD)	X’Pert Pro	Siweihechuang Technology Co., Ltd., Wuhan, China.
Scanning Electron Microscope (SEM)	SU8220	Hisense Hitachi Air Conditioning System Co., Ltd., Qingdao, China.
Laser particle size analyzer	TopSizer	Nanoparticle Instrument Co., Ltd., Jinan, China.
Argon ion cutting machine	IB-19530CP	Rui Ling Industrial Group Co., Ltd., Shenzhen, China.
Glove box	MKUS2-1908-0100	Zhongke Guangzhi Technology Co., Ltd., Chongqing, China.
Blue Power Testing System	CT 2001A	Deep Blue Power Technology Co., Ltd., Wuhan, China.
Analysis balance	BS214S	Sedoli Automation Technology Co., Ltd., Dongguan, China.
Electrochemical workstation	CHI 660E	Chenhua Technology Co., Ltd., Shanghai, China.
Coating machine	MSK-AFA-ES200	Kejing Zhida Technology Co., Ltd., Shenzhen, China.
Snap-type battery slicer	MSK-T10	Kejing Zhida Technology Co., Ltd., Shenzhen, China.
Electric twin-roll mill	MSK-2150	Kejing Zhida Technology Co., Ltd., Shenzhen, China.
Electric sealing machine	MSK-E110	Kejing Zhida Technology Co., Ltd., Shenzhen, China.
High-speed disperser	ARM-310	Kejing Zhida Technology Co., Ltd., Shenzhen, China.
Electric heating constant-temperature forced-air drying oven	DHG-1024	Yiheng Scientific Instrument Co., Ltd., Shanghai, China.
ICP	ICP6600	Agilent Technologies, Inc., Santa Clara, CA, USA.

**Table 3:** The content of impurity elements in the three types of silicon raw materials.

Test Data	Impurity Elements (ppm)	Purity (%)
Monocrystalline silicon	<20	99.98
High-purity silicon	<10	99.99
Silicon metal	<10	99.99

## Data Availability

The original contributions presented in this study are included in the article. Further inquiries can be directed to the corresponding author.
